# Potent New Small-Molecule Inhibitor of Botulinum Neurotoxin Serotype A Endopeptidase Developed by Synthesis-Based Computer-Aided Molecular Design

**DOI:** 10.1371/journal.pone.0007730

**Published:** 2009-11-10

**Authors:** Yuan-Ping Pang, Anuradha Vummenthala, Rajesh K. Mishra, Jewn Giew Park, Shaohua Wang, Jon Davis, Charles B. Millard, James J. Schmidt

**Affiliations:** 1 Computer-Aided Molecular Design Laboratory, Mayo Clinic, Rochester, Minnesota, United States of America; 2 Division of Biochemistry, Walter Reed Army Institute of Research, Silver Spring, Maryland, United States of America; 3 Integrated Toxicology Division, United States Army Medical Research Institute of Infectious Diseases, Frederick, Maryland, United States of America; Leeds Institute of Molecular Medicine, United Kingdom

## Abstract

Botulinum neurotoxin serotype A (BoNTA) causes a life-threatening neuroparalytic disease known as botulism. Current treatment for post exposure of BoNTA uses antibodies that are effective in neutralizing the extracellular toxin to prevent further intoxication but generally cannot rescue already intoxicated neurons. Effective small-molecule inhibitors of BoNTA endopeptidase (BoNTAe) are desirable because such inhibitors potentially can neutralize the intracellular BoNTA and offer complementary treatment for botulism. Previously we reported a serotype-selective, small-molecule BoNTAe inhibitor with a *K*_i_^app^ value of 3.8±0.8 µM. This inhibitor was developed by lead identification using virtual screening followed by computer-aided optimization of a lead with an IC_50_ value of 100 µM. However, it was difficult to further improve the lead from micromolar to even high nanomolar potency due to the unusually large enzyme-substrate interface of BoNTAe. The enzyme-substrate interface area of 4,840 Å^2^ for BoNTAe is about four times larger than the typical protein-protein interface area of 750–1,500 Å^2^. Inhibitors must carry several functional groups to block the unusually large interface of BoNTAe, and syntheses of such inhibitors are therefore time-consuming and expensive. Herein we report the development of a serotype-selective, small-molecule, and competitive inhibitor of BoNTAe with a *K*_i_ value of 760±170 nM using synthesis-based computer-aided molecular design (SBCAMD). This new approach accounts the practicality and efficiency of inhibitor synthesis in addition to binding affinity and selectivity. We also report a three-dimensional model of BoNTAe in complex with the new inhibitor and the dynamics of the complex predicted by multiple molecular dynamics simulations, and discuss further structural optimization to achieve better *in vivo* efficacy in neutralizing BoNTA than those of our early micromolar leads. This work provides new insight into structural modification of known small-molecule BoNTAe inhibitors. It also demonstrates that SBCAMD is capable of improving potency of an inhibitor lead by nearly one order of magnitude, even for BoNTAe as one of the most challenging protein targets. The results are insightful for developing effective small-molecule inhibitors of protein targets with large active sites.

## Introduction

Botulism is a life-threatening neuroparalytic disease that occurs in at least five forms–food-borne botulism, infant botulism, wound botulism, adult enteric infectious botulism, and inhalation botulism. The disease is caused by the specific action of botulinum neurotoxins. Seven antigenically distinct botulinum neurotoxins (serotypes A to G) are known, but primarily serotypes A, B, E, and F have been reported to cause botulism in humans.

Botulinum neurotoxin serotype A (BoNTA) is a protein produced by the spore-forming anaerobic bacterium, *Clostridium botulinum*. The lethal doses of BoNTA for a 70-kg human are estimated as 0.9 µg by inhalation and 70 µg by ingestion [1]. BoNTA inhibits the release of acetylcholine from presynaptic nerve terminals at neuromuscular junctions, causing flaccid paralysis and frequently leading to prolonged mechanical ventilation with serious medical sequelae or death following respiratory arrest [2]. Despite its toxicity, the purified and diluted BoNTA has been used as medical treatment for cholinergic nerve and muscle dysfunctions [3], [4]; BoNTA (marketed as Botox) also can temporarily reduce facial frown lines. However, overdose with BoNTA can develop systemic botulism [5].

Current post-exposure treatment of botulism relies on administration of antibodies against the toxins. The available antitoxin is an equine product that causes allergic reactions in ∼10% of patients [6]. In late 2003, a solution of human antibodies was licensed as “BabyBIG” to treat infant botulism [7], but to date generic botulism immune globulin injections are still unavailable. Furthermore, antibodies are effective in neutralizing the extracellular toxin to prevent further intoxication but generally cannot rescue already intoxicated neurons. There is a need to develop complementary, post-exposure treatments such as small-molecule antidotes that potentially can neutralize the intracellular toxin.

**Figure 1:**
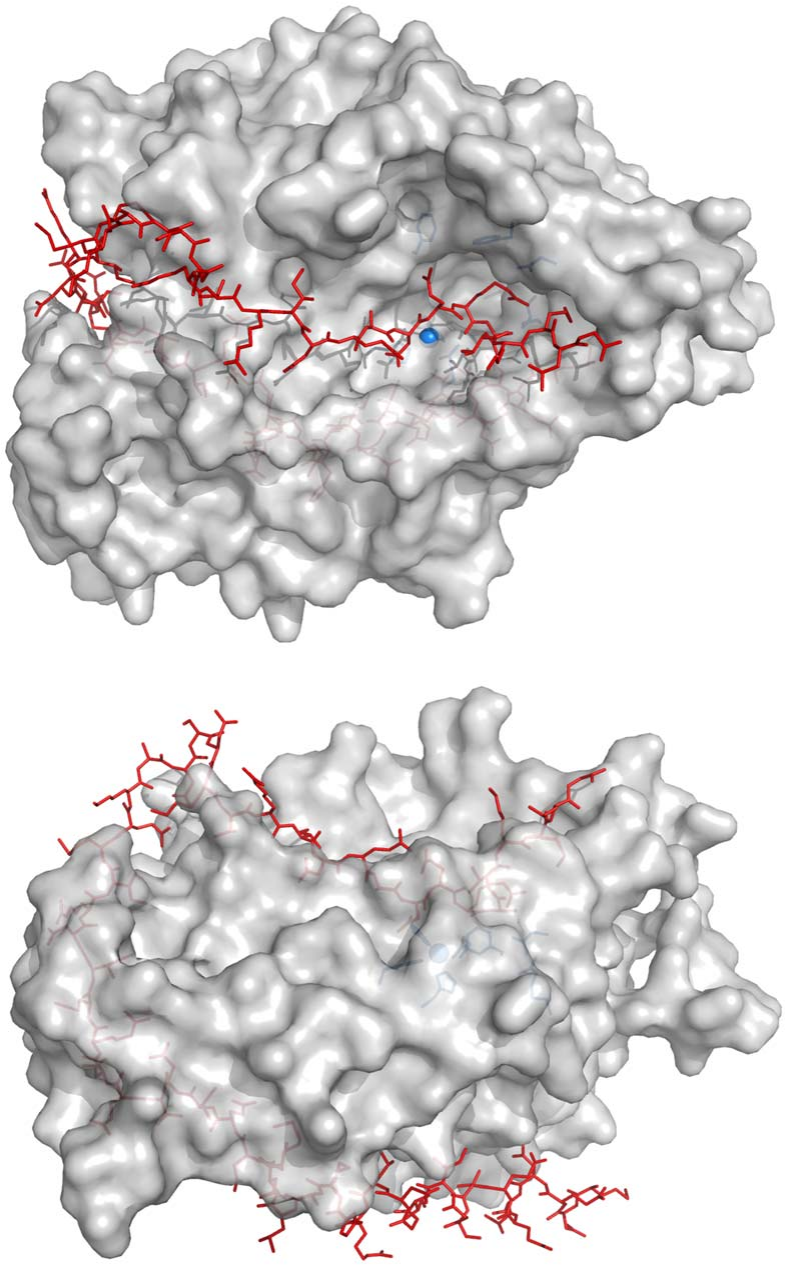
Large enzyme-substrate interface of BoNTAe. A: top view of the active site showing the substrate binding at the large pocket. B: side view of the active site showing the substrate wrapping around the circumference of BoNTAe. Active-site residues of BoNTAe (Zn^+2^, H223, H227, E262, F163, F194, R363, and D370) are shown in light blue sphere or light blue stick model. The SNAP-25 substrate (146–204) is shown in red stick model. BoNTAe is shown in grey surface model with 15% transparency.

Structurally, BoNTA consists of a light chain (M_r_ ∼50,000) and a heavy chain (M_r_ ∼100,000) that are linked by a disulfide bond. The light-chain is a zinc endopeptidase that specifically cleaves SNAP-25, a neuronal protein required for acetylcholine release [8]. The BoNTA endopeptidase (BoNTAe) has been a target for developing small-molecule inhibitors of the toxin [9]–[15]. However, developing potent inhibitors is as difficult as developing small-molecule inhibitors of protein-protein complexes. The latter has been a known challenge for decades [16]. The difficulty arises because, upon binding, the substrate wraps around the circumference of BoNTAe constituting an unusually large substrate enzyme interface area of 4,840 Å^2^ (Figure 1) [17]. This interface requires molecules possessing several functional groups to block it. Consequently, syntheses of such molecules are time-consuming and costly. The extent of the challenge can be appreciated by comparing the interface area (4,840 Å^2^) of BoNTAe to the typical protein-protein interface area of 750–1500 Å^2^ [16]. Because of the large-interface challenge, small-molecule BoNTAe inhibitors reported to date typically have inhibitory potencies in the low micromolar range (see Section 3.1). To date, the most potent BoNTAe inhibitor has been an elegantly designed peptidic analog that mimics GlnArgAlaThrLysMetLeu, and this heptapeptidic mimetic (**HPM**) has a *K*_i_ value of 41 nM [18]. Nevertheless, some of the reported micromolar small-molecule inhibitors already demonstrate *ex vivo* efficacy in protecting cells against BoNTA [9], [11]. Our micromolar small-molecule BoNTAe inhibitor leads also demonstrate *in vivo* efficacy at a concentration of 2 mg/kg in protecting mice against 5 LD_50_ challenge of BoNTA (manuscript in preparation). These results suggest that BoNTAe is a viable target for developing small-molecule antidotes to BoNTA.

Previously we reported a serotype-selective, small-molecule BoNTAe inhibitor with a *K*_i_^app^ value of 3.8±0.8 µM [10]. This inhibitor was developed by lead identification using virtual screening followed by optimization of a lead with an IC_50_ value of 100 µM [19]. However, we were unable to further improve the lead from micromolar to even high-nanomolar potency using conventional computer-aided molecular design (CAMD), despite the use of a high-resolution BoNTAe crystal structure (free and in complex with a peptidic substrate analog) [20] and advanced terascale computer simulation techniques [10].

In this article, we report the development of a serotype-selective, small-molecule, and competitive inhibitor of BoNTAe with a *K*_i_ value of 760±170 nM by using a new approach, termed synthesis-based computer-aided molecular design (SBCAMD), to address the challenge in synthesis. We also report a three-dimensional (3D) model of the high-nanomolar inhibitor in complex with BoNTAe and the dynamics of the complex predicted by multiple molecular dynamics simulations (MMDSs). On the basis of the 3D model, we discuss insights into further structural optimization to achieve *K*_i_ values comparable to that of **HPM** and the prospect of small-molecule BoNTAe inhibitors as effective antidotes to neutralize the intracellular BoNTA. We also discuss the advantages of both SBCAMD and CAMD in drug discovery.

## Results

### Inhibitor Design

**Figure 2:**
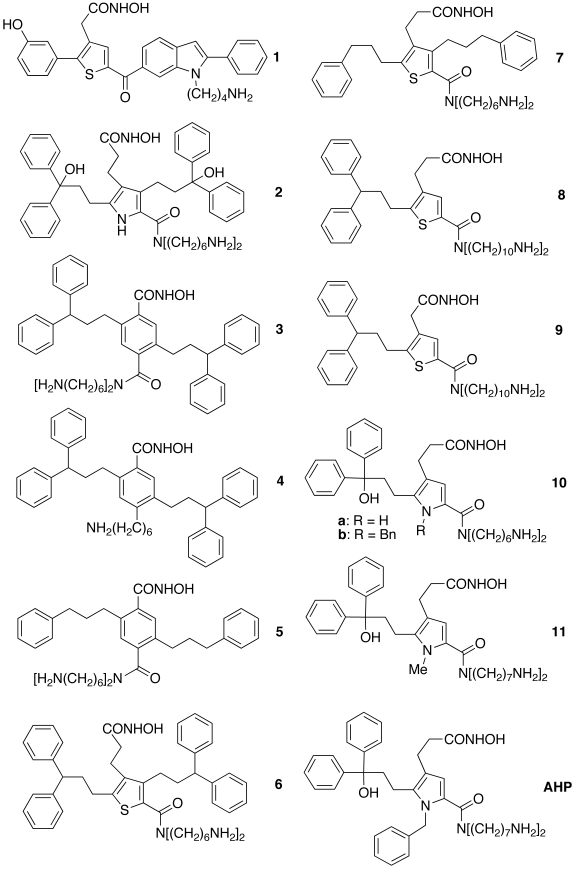
Chemical structures of BoNTAe inhibitors and their analogs resulted from SBCAMD.

MMDSs on our early lead **1** (Figure 2) [10] suggested that structures like analog **2** (Figure 2) have higher affinities for BoNTAe than **1**. This is because (1) one diphenylmethanol group of **2** can adopt the Venus-flytrap-like conformation to grasp Arg363 of BoNTAe more tightly via cation-pi interactions than the phenyl group of **1**; (2) the other diphenylmethanol group can fill the large void of the BoNTAe active site and interact with Arg231 via cation-pi interactions, whereas **1** cannot reach Arg231; (3) two ammonium groups of **2** rather than only one ammonium group of **1** can interact with four carboxylates (Glu55, Glu64, Glu164, and Glu257) at the active site.

However, we could not make **2** using the scheme shown in Figure S1 in a short period, nor could we make analog **6** (Figures 2 and S2), a thiophene analog of **2**, given the same time constraint. We subsequently replaced the pyrrole ring of **2** with a benzene ring to avoid the problem of making **2**. This transformation resulted in analogs **3–5** (Figure 2) that can be made with facile syntheses described in the schemes shown in Figure S3, Figure S4, and Figure S5, although these molecules are more bulky than **2** and carry an aromatic hydroxamate that has a relatively low affinity for zinc due to pi-electron delocalization. We initially hoped that the bulkiness problem could be remedied by the flexibility of the BoNTAe active site and therefore made **3–5** with ease. However, biological evaluation showed that none of these analogs was active in inhibiting BoNTAe at an inhibitor concentration of 10 µM (Table 1). Although change of an aromatic hydroxamate to an aliphatic hydroxamate does not require too much synthetic effort, we did not pursue this modification, as it would make these molecules even more bulky.

**Table 1:** Relative BoNTAe inhibition activities of AHP and its analogs.

Inhibitor	% Inhibition of BoNTAe
**1**	46% at 10 µM
**2**	Not made
**3**	0% at 10 µM
**4**	3% at 10 µM
**5**	1% at 10 µM
**6**	Not made
**7**	Precipitated in assay buffer
**8**	35% at 10 µM
**9**	35% at 10 µM
**10a**	Not made
**10b**	64% at 10 µM
**11**	8% at 1 µM
**AHP**	54% at 1 µM

**Figure 3:**
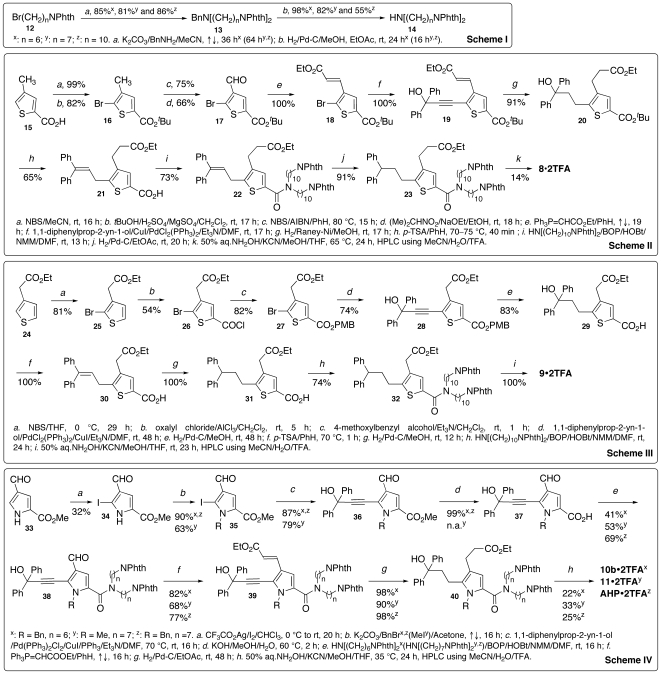
Syntheses of BoNTAe inhibitors 8, 9, 10b, 11, and AHP. Scheme I: synthesis of intermediate **14**; Scheme II: synthesis of **8**; Scheme III: synthesis of **9**; Scheme IV: syntheses of **10b**, **11**, and **AHP**.

Alternatively, we made analog **7** (Figure 2) as a truncated analog of **6** according to the scheme shown in Figure S6. Unexpectedly, **7** precipitated in the inhibition assay buffer even though dimethyl sulfoxide was used to dissolve **7**. Gratifyingly, a different truncation of **6** led to the syntheses of analogs **8** and **9** (Figure 2) according to Schemes I–III of Figure 3. Encouragingly, both **8** and **9** showed 35% inhibition of BoNTAe at an inhibitor concentration of 10 µM (Table 1). These results inspired the design of analog **10a** (Figure 2). The replacement of the thiophene ring of **8** by a pyrrole ring in **10a** was due to our synthetic experience and available intermediates of the unsuccessful synthesis of **2**. In making **10a** according to Schemes I and IV of Figure 3, we had to protect the pyrrole nitrogen atom in order to improve the yield of the Sonagashira coupling [21]. However, we could not cleave the *p*-methoxybenzyl or benzyl group from the pyrrole nitrogen atom, nor could we protect the nitrogen atom using tosyl chloride. Nonetheless, according to the inhibitor-bound BoNTAe complexes generated by MMDSs, we conjectured that the benzyl group of **10b** could interact favorably with the serotype-specific Phe194 via the pi-pi interaction. We therefore completed the synthesis of **10a** without cleaving the benzyl group to give **10b**. More encouragingly, **10b** at 10 µM showed 64% inhibition of BoNTAe (Table 1). A structure-activity relationship study on a series of homologues of **10b** with two identical alkylamino chain lengths varying from 6 to 10 methylene groups identified *N*,*N*-bis(7-aminoheptyl)-1-benzyl-5-(3-(hydroxy-3,3-diphenylpropyl)-4-(3-hydroxyamino)-3-oxopropyl))-1*H*-pyrrole-2-carboxamide (**AHP**, see Figure 2) as the most potent of the series (see Section 2.4 for characterization of **AHP**). To confirm the conjecture about the interaction of the benzyl group with Phe194, we also made analog **11** with a methyl group attached to the pyrrole nitrogen atom of **11** (Figures 2 and 3). Indeed, **11** at 1 µM showed only 8% inhibition of BoNTAe, whereas **AHP** exhibited 54% inhibition under the same conditions (Table 1).

### Computer Simulation

Twenty different molecular dynamics simulations (each lasted 10 ns) were carried out for the **AHP**•BoNTAe complex to probe the intermolecular interactions of the design inhibitor using the PMEMD module of the AMBER 8 program [22] (see Section 4.4). The initial complex structure was generated by manually docking **AHP** into the BoNTAe active site with (1) the hydroxamate group near the active-site zinc ion, (2) the diphenylmethanol group near Arg363, (3) the benzyl group near Phe194, (4) one ammonium group near Glu55 and Glu164, and (5) the other ammonium group near Glu257. The BoNTAe structure used for the docking was taken from the crystal structure of an inhibitor-bound BoNTAe (Protein Data Bank Code: 3BOO [20]) in which conformations of missing residues 62–67 were taken from the crystal structure of a BoNTAe mutant in complex with SNAP-25 (Protein Data Bank Code: 1XTG [17]).

For each of the 20 simulations, 200 instantaneous conformations were saved at 5-ps intervals during the last 1-ns period. A total of 4,000 instantaneous conformations of **AHP**•BoNTAe from the 20 simulations were subjected to a cluster analysis using the averagelinkage algorithm (epsilon = 2.0 Å and RMS on alpha-carbon atoms) [23] implemented in the PTRAJ module of the AMBER 10 program [22]. Only one cluster of the BoNTAe conformations was identified.

**Figure 4:**
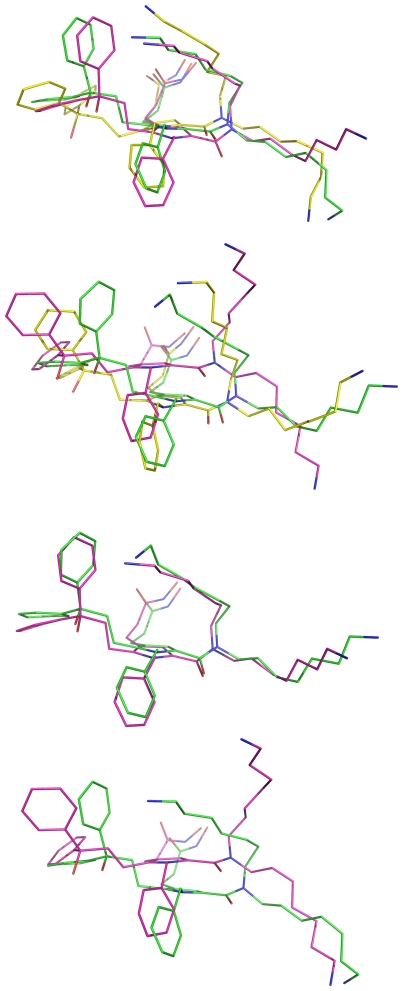
Overlays of AHP conformations of the three most populated clusters from the MMDSs. A (row 1): Clusters 1 (green), 2 (magenta), and 3 (yellow) from the first-round; B (row 2): Clusters 1 (yellow), 3 (green), and 5 (magenta) from the second-round; C (row 3): Cluster 2 (magenta) of the first-round and Cluster 3 (green) of the second-round; D (row 4): Cluster 1 (green) of the first-round and Cluster 5 (magenta) of the second-round.

All 4,000 instantaneous conformations of **AHP**•BoNTAe were subjected to a second-round cluster analysis using the averagelinkage algorithm (epsilon = 2.0 Å and RMS on all atoms of **AHP**). This analysis identified five clusters of the **AHP** conformations. The numbers of the **AHP** conformations in Clusters 1–5 are 2400, 600, 721, 200, and 79, respectively. Of the three most populated clusters (Figure 4), the **AHP** conformations in Clusters 1 and 3 are similar, and the one in Cluster 2 is unique. All inhibitor conformations in Clusters 1–3 have one ammonium group forming a salt bridge to Asp370, although this group was placed away from Asp370 but close to Glu257 in the initial conformation used in the MMDSs. For the inhibitor conformations in Cluster 2, the other ammonium group moved away from Glu56 and Glu164, and the ammonium group of Lys66 moved close to the two carboxylates to form salt bridges, although the ammonium groups of **AHP** and Lys66 were placed close to and away from the carboxylates in the initial conformation, respectively. For the inhibitor conformations in Clusters 1 and 3, the Glu56-interacting ammonium group of **AHP** and the Lys66 ammonium group remained in the same locations as those in the initial conformation.

To investigate whether **AHP** adopts two different conformations found in Clusters 1 and 2 or one of the two, a second round of 20 10-ns-long simulations on **AHP**•BoNTAe were carried out using the representative conformation of **AHP**•BoNTAe in Cluster 2 as an initial conformation. The same cluster analyses of the second-round simulations identified again one cluster of the BoNTAe conformations and five clusters of the **AHP** conformations. The numbers of the inhibitor conformations in Clusters 1–5 of the second-round simulations are 459, 201, 2762, 178, and 400, respectively. Interestingly, the most populated inhibitor conformation (Cluster 3) of the second-round simulations is nearly identical to the third most popular inhibitor conformation (Cluster 2) of the first-round. However, the most popular inhibitor conformation (Cluster 1) of the first-round simulations disappeared in the second-round, suggesting that **AHP** adopts only one conformation in the BoNTAe active site.

**Figure 5:**
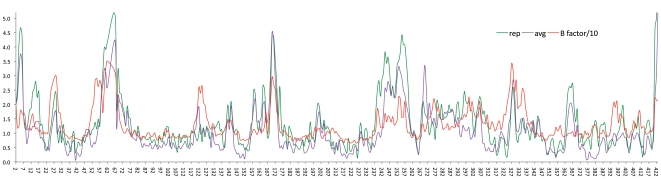
Deviations for alpha carbons between the BoNTAe crystal structure and the aligned MMDS-generated AHP•BoNTAe and B factors for the corresponding alpha carbons of the crystal structure. The crystal structure was taken from Protein Data Bank (Code: 3BON [20]). The representative (rep) and average (avg) conformations of **AHP**•BoNTAe were obtained from the second-round MMDSs. Horizontal axis: residue ID; vertical axis: alpha-carbon deviation in Å and B factors scaled by 10% in Å^2^.

Deviations for all alpha-carbon atoms between the representative conformation of Cluster 3 of the second-round simulations and the free BoNTAe crystal structure (Protein Data Bank Code: 3BON [20]) that was aligned over the representative conformation were computed and plotted against temperature factors of the corresponding alpha-carbon atoms (Figure 5). A similar plot was also obtained from the average conformation of Cluster 3 of the second-round simulations (Figure 5). It is worth noting that only alpha-carbon atoms were used for the alignment and that no energy minimization was performed on the representative or average conformation. As apparent from these correlation plots, the alpha-carbon deviations of the representative and average conformations are consistent with temperature factors for all residues except for terminal residues or residues that are involved in crystal contact such as residues 197–213, 244–257, 283–295, and 375–387. The representative conformation of **AHP**•BoNTAe in Cluster 3 of the second-round simulations has a free-and-cool root mean square deviation (C&F RMSD) of 1.06 Å for all alpha-carbon atoms of the protein relative to those of the crystal structure. The C&F RMSD [24] is the RMSD of residues that are not involved in crystal contact and have temperature factors lower than the average temperature factors, and it is used to minimize the structural difference caused by crystal packing [25]–[27] and high temperature factors. Consistently, the average conformation of **AHP**•BoNTAe in Cluster 3 of the second-round simulations has a C&F RMSD of 0.85 Å. These results suggest that the average or representative conformation in Cluster 3 of the second-round simulations best represents the **AHP**•BoNTAe complex structure in water. The coordinates of the average and representative conformations are available from Datasets S1 and S2 of Supporting Information.

**Figure 6:**
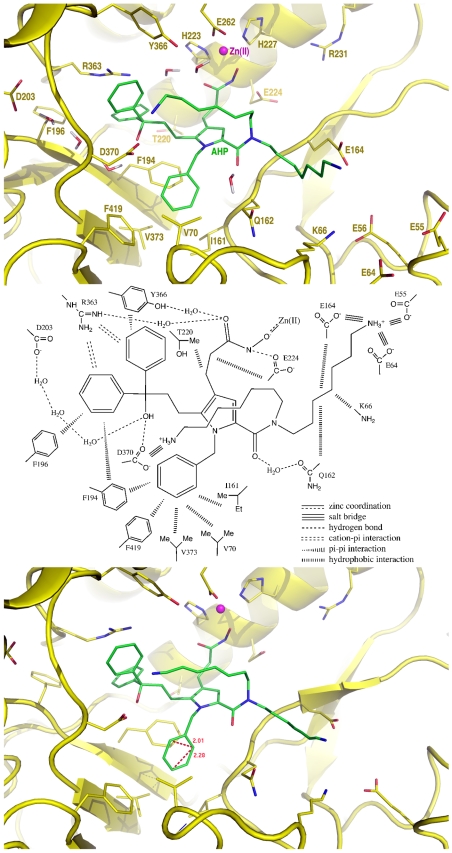
Close-up view of the intermolecular interactions of AHP with the active-site residues of BoNTAe. A (top): representative conformation generated by the second-round MMDSs; B (middle): diagram of the intermolecular interactions between **AHP** and BoNTAe; C (bottom): average conformation generated by the second-round MMDSs.

In the representative conformation of **AHP**•BoNTAe (Figures 6A and 6B), the hydroxamate group coordinates the zinc ion and has (1) a hydrogen bond to Glu224, (2) a one-water-mediated hydrogen-bond network to Tyr366, and (3) a one-water-mediated hydrogen-bond network to Arg363; the two hydroxamate-linking methylene groups have van der Waals interactions with the methyl and methylene groups of Thr220 and Glu224, respectively; the diphenylmethanol group has (1) cation-pi interactions with Arg363, (2) pi-pi interactions with Tyr366, Phe194, and Phe196, (3) a hydrogen bond to Asp370, and (4) a three-water-mediated hydrogen-bond network to Asp203; the benzyl group has pi-pi interactions with Phe194 and Phe419; the pyrrole-substituted carbonyl oxygen atom has a one-water-mediated hydrogen-bond network to Gln162; one ammonium group has a salt bridge with Asp370 and an intramolecular cation-pi interaction with the diphenylmethanol group; the other ammonium group is placed at the mid point to the carboxylates of Glu55, Glu64, and Glu164 and has ionic interactions with these carboxylates; the alkyl chain of the Glu55-interacting ammonium group has van der Waals interactions with the methylene groups of Glu164, Lys66, and Gln162.

In the average conformation of **AHP**•BoNTAe (Figure 6C), there is significant contraction for the benzyl group and the alkyl ammonium group that interacts with Glu55, Glu64, and Glu164. The contraction of the benzyl group indicates that it spins in a hydrophobic pocket constituted by Val70, Ile161, Phe194, Val373, and Phe419. It also indicates that the alkyl ammonium group is highly mobile in interacting with Glu55, Glu64, and Glu164. The inhibitor dynamics revealed by the MMDSs suggests that the binding of **AHP** to BoNTAe is partly entropy-driven. It also suggests that the electron density for the benzyl group will be slightly disordered and that the electron density for one alkyl ammonium group will be significantly disordered in the crystal structure of **AHP**•BoNTAe to be determined.

### Chemical Synthesis

**AHP**, an *achiral* molecule possessing five functional groups (hydroxamate, diphenylmethanol, benzyl, and two ammonium groups) designed to interact with the active-site residues of BoNTAe, was made readily with a 10-step synthesis outlined in Schemes I and IV of Figure 3. This synthesis began with methyl 4-formyl-1*H*-pyrrole-2-carboxylate (**33**) that has a pyrrole ring as a frame to support the five functional groups in different orientations. This expensive commercially available starting material was, however, made in-house from 2-(trichloroacetyl)pyrrole using a two-step synthesis according to published protocols [28], [29]. To introduce the diphenylmethanol group to the pyrrole ring via the Sonagashira coupling [21], I_2_ and CF_3_CO_2_Ag in CHCl_3_ [29] were used to iodinate intermediate **33** to give the desired intermediate **34**, which was separable from methyl 4-formyl-3,5-diiodo-1*H*-pyrrole-2-carboxylate and methyl 4-formyl-3-iodo-1*H*-pyrrole-2-carboxylate through column chromatography. Another reported procedure [30] was also effective in converting **33** to **34** in a similar yield. *N*-Benzylation of **34** followed by the Sonagashira coupling [21] with 1,1-diphenylprop-2-yn-1-ol gave intermediate **36x** with both diphenylmethanol and benzyl groups linked to the pyrrole ring, which was confirmed by the heteronuclear multiple bond coherence (HMBC) spectroscopy. Saponification of **36x** followed by amidation using intermediate **14y** gave intermediate **38z** with two protected alkylamino groups linked to the pyrrole ring, wherein **14y** was obtained from *N*-(7-bromoheptyl)-phthalimide in a high yield via a two-step synthesis outlined in Scheme I of Figure 3. Wittig olefination of **38z** gave intermediate **39z** with an ethyl ester as a precursor of the desired hydroxamate group. The two unsaturated bonds of **39z** were reduced by dry 10% Pd-C in ethyl acetate to give intermediate **40z**. The hydrogenation reaction was very sensitive to moisture; wet Pd-C or Raney nickel resulted in unidentified products. The final product (**AHP**) was obtained by treating **40z** with excess of 50% aqueous hydroxylamine for 24 hours [31]. This treatment converted the ethyl ester and *N-*phthalimides to hydroxamic acid and amines, respectively [32], [33]. High performance liquid chromatography (HPLC) purification using TFA-containing solvent yielded pure **AHP** in its TFA salt form. Caution should be taken in removing TFA-containing eluent after HPLC, as the diphenylmethyl hydroxy group of **AHP** is prone to elimination under acidic conditions (see Section 4.2.7).

### Biological Testing

**Figure 7:**
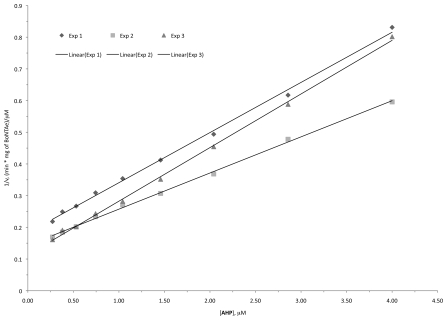
Dixon plots for competitive inhibition of BoNTAe by AHP.

According to HPLC-based kinetics assays described in Section 4.3 [34], **AHP** has a *K*_i_ value of 760±170 nM, which was calculated from slopes of Dixon plots (Figure 7) with *K*_i_ = *K*_M_/[(slope)(V_MAX_)(S)], where (S) is the substrate concentration [35] and kinetic constants for the substrate were taken from reference [36]. Unlike inhibitor **1** as we reported previously [10], **AHP** has linear Dixon plots (Figure 7) indicating that **AHP** is a competitive inhibitor of BoNTAe and its *K*_i_ value can thus be obtained from the fractional inhibition method or the Cheng and Prusoff method [35], [37]. Indeed, both methods yielded a *K*_i_ value of 760±170 nM, consistent with the value from the Dixon plots. **AHP** did not inhibit botulinum neurotoxin serotype B endopeptidase (BoNTBe) at concentrations up to 5 µM at which **AHP** showed >80% inhibition of BoNTAe. As described in Section 4.3, the BoNTAe inhibition assay buffer was supplemented by 25 µM ZnCl_2_, whereas the BoNTBe inhibition assay buffer contained no exogenous zinc ion. **AHP** at 5 µM showed >80% inhibition of BoNTAe in the presence of 5-molar excess of exogenous zinc, but it did not show any BoNTBe inhibition in the absence of exogenous zinc. These results preclude the possibility that **AHP** nonspecifically inhibited BoNTAe through depleting the active-site zinc divalent cation via zinc chelation. Clearly, **AHP** is a serotype-selective, small-molecule, and competitive inhibitor of BoNTAe with a *K*_i_ value of 760±170 nM.

## Discussion

### Relative Potencies of BoNTAe Inhibitors

A mixture of two regioisomeric small molecules has been reported to have a *K*_i_ value of 600±100 nM in inhibiting BoNTAe [38]; however, there has been no report for the separation of the mixture. To our knowledge, the most potent BoNTAe inhibitor has been **HPM**, a peptidic inhibitor with a *K*_i_ value of 41 nM [18]. A small-molecule BoNTAe inhibitor, 2,4-dichlorocinnamic hydroxamate (**DCCH**), was reported to have initially a *K*_i_ value of 300±12 nM [39] and most recently an IC_50_ value of 59 or 81 µM depending on assay conditions [40]. Using the assay conditions described in Section 4.3 that are different from those reported in [39], [40], we found that the IC_50_ values of **AHP** and **DCCH** are <1 µM and 34 µM, respectively, and that **AHP** is >34-fold more potent in inhibiting BoNTAe than **DCCH** [39] and one order of magnitude less potent than **HPM** [18]. Given this rank order of potency, below we discuss new insights into BoNTAe inhibitor design and new approaches that facilitate such design.

### Insights into BoNTAe Inhibitor Design

While crystal structures of **HPM**•BoNTAe and **DCCH**•BoNTAe have been reported [18], [41], the determination of the crystal structure of **AHP**•BoNTAe is currently underway. The results described in Section 2.2 suggest that the computer model of **AHP**•BoNTAe is suitable for qualitative comparison of the binding of **AHP** at the BoNTAe active site with those of **HPM** and **DCCH**.

**Figure 8:**
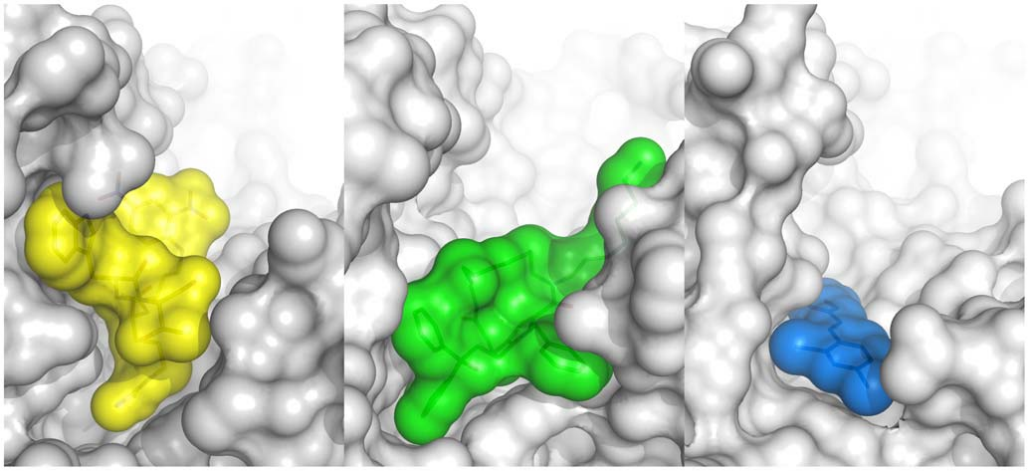
Cross-Section Views of HPM, AHP, and DCCH bound in the BoNTAe active site. Left: **HPM** with carbon in yellow obtained from the crystal structure of 3DS9 [18]; middle: **AHP** with carbon in green obtained from the MMDS-generated model; right: **DCCH** with carbon in blue obtained from the crystal structure of 2IMA [41]. Oxygen, nitrogen, and zinc are shown in red, blue, and light blue, respectively. BoNTAe is shown in white surface model with 15% transparency.

As apparent from the cross-section view of the inhibitor-bound BoNTAe active site (Figure 8), **DCCH** occupies a small portion of the active-site region where the Arg198-containing hydrolysis product binds [17]; **AHP** occupies the bottom half of that region; **HPM** occupies nearly the entire region. The rank order of occupancy in the active site for the three inhibitors agrees well with the rank order of the inhibitory potency described in Section 3.1. From structural point of view, this agreement suggests that effective BoNTAe inhibitors have volumes that are comparable to or more than that of **AHP**. The “bulkiness” requirement observed is consistent with the reported nanomolar inhibitors of BoNTBe [42]. Therefore, we anticipate that optimization of known small-molecule BoNTAe inhibitors by small structural perturbations alone is unlikely to lead to effective BoNTAe inhibitors.

To obtain more potent nanomolar small-molecule BoNTAe inhibitors, we think that new functional groups have to be introduced to **AHP** to occupy the upper portion of the active-site region. However, the more functional groups are introduced, the more challenging the inhibitor synthesis will become. In addition, adding more functional groups can hamper cell permeability. For these reasons, sophisticated computer simulations such as MMDSs employing the cationic dummy atom approach to simulating zinc proteins [19], [43]–[45] are needed to identify new functional groups. These groups must have high affinities for the BoNTAe active site and low molecular weights to maintain cell permeability. In our view, for a realistic prospect of obtaining nanomolar BoNTAe inhibitors able to antagonize the intracellular BoNTA, the design and optimization of current BoNTAe inhibitor leads may need new approaches such as the nonbonded bivalence approach [46] that dimerizes composite inhibitors by intermolecular interactions (self-assembly) rather than covalent bonds in order to improve both affinity and permeability.

### Synthesis-Based Computer-Aided Molecular Design

Despite the use of high-performance computing to predict inhibitor affinity and serotype selectivity in developing our small-molecule BoNTAe inhibitors, we recognized that the development process is still a *brute force* process for at least two reasons. First, exquisitely designed inhibitors such as inhibitor **2** may not be synthetically accessible within a reasonable amount of time. Second, like analog **7**, newly made inhibitors may not dissolve in an assay buffer under a high ionic strength condition. Identification of these problems *a priori* is beyond the scope of current computer simulations. The best way to circumvent these problems is to make and test more inhibitors with good affinities predicted by computer simulations. This requires facile synthesis of more of such inhibitors, which is, however, different from high-throughput screen of any available chemicals. Therefore, the ability to obtain nanomolar BoNTAe inhibitors relies more on expeditious syntheses of inhibitor analogs with good affinities than on exquisite design of high-affinity inhibitor analogs using free energy perturbation calculations that can compute the absolute binding free energy [10].

With these considerations, we pursued synthesis-based computer-aided molecular design (SBCAMD) that accounts the practicality and efficiency of inhibitor synthesis in addition to binding affinity and selectivity. The general form of our proposed approach comprises (1) identification of an inhibitor lead with the aid of computer simulations (*e.g.*, identifying **2** by MMDSs); (2) transformation of the lead to molecules that can be made in a reasonable amount of time (*e.g.*, converting **2** to inhibitors **5–10**); (3) re-evaluation of the synthetically accessible molecules with computer simulations (*e.g.*, confirming the intermolecular interactions of **AHP** through MMDSs), this step may not be necessary if inhibitor synthesis takes less time than computer simulations; (4) synthesis and evaluation of the molecules from Step 2 or 3 (*e.g.*, synthesizing and testing **AHP**).

From synthetic chemistry point of view, SBCAMD uses a facile synthetic scheme to transform commercially available or known chemicals into a molecule that fits or likely fits the binding site of a drug target. In this case, the target molecule is determined with consideration of its synthetic effort. In contrast, CAMD uses retrosynthetic analysis [47] to repeatedly transform a target molecule as a potential inhibitor of a drug target into simpler precursors until commercially available or known molecules are reached. In this case, like a natural product, the target molecule is pre-determined regardless of its synthetic effort.

SBCAMD is proposed to work with large-binding-pocket targets, while CAMD works best with small-binding-pocket targets. There are knowingly a number of factors (such as solubility in an assay buffer) that are beyond the scope of current computer simulations, but govern the potency of an inhibitor. Therefore, the more inhibitors are tested, the more likely a better inhibitor can be identified. The CAMD approach works well with small-binding-pocket targets, even though it does not address practicality and efficiency of chemical synthesis for each “computer-identified” inhibitor to be tested. This is because inhibitors for this kind of targets are structurally simple and often commercially available or can be made without too much synthetic effort. When bulky inhibitors are needed to bind a large protein pocket, such inhibitors are structurally complex and generally commercially unavailable. Therefore, inhibitor syntheses inevitably become the rate determine step of the development process, and hence SBCAMD comes into play.

More case studies are certainly needed, however, in our view, SBCAMD holds promise in developing small-molecule BoNTAe inhibitors or other small-molecules inhibitors that bind at large interfaces, because it aims to reduce the number of chemicals to be tested relative to the number of chemicals used in the high throughput screen and the time and cost to make each chemical to be tested.

### Conclusion

We developed a serotype-selective, small-molecule, and competitive inhibitor of BoNTAe with a *K*_i_ of 760±170 nM. We also developed a 3D model of BoNTAe in complex with the inhibitor, which supports the concept underlying the design of the inhibitor. These developments were made possible by the use of a new approach (SBCAMD) that aims to reduce the number of chemicals to be tested as well as the time and cost to make each chemical to be tested. The results provide insights into developing effective small molecules that can neutralize the intracellular BoNTA to provide a therapy complimentary to current antibody therapy for treating botulinum. This work offers an example of the use of SBCAMD to improve expeditiously the potency of an inhibitor lead by nearly one order of magnitude for BoNTAe that is arguably one of the most challenging protein targets.

## Materials and Methods

### Reagents

Hexanes (Hex), ethyl acetate (EtOAc), and TFA were purchased from Fisher Scientific (Pittsburgh, PA) and used without purification. CF_3_CO_2_Ag and 1,1-diphenylprop-2-yn-1-ol were purchased from Aldrich Chemical Co Inc (Milwaukee, WI). Dry Pd-C catalyst was purchased from Strem Chemicals Inc (Newburyport, MA). All other commercially available reagents were used as received. Recombinant BoNTAe was provided by Dr. Leonard Smith of the United States Army Medical Research Institute of Infectious Diseases, Fort Detrick, MD. BSA, HEPES buffer, and zinc chloride were purchased from Sigma-Aldrich (St. Louis, MO). Dithiothreitol was obtained from BioRad (Hercules, CA).

### Chemical Synthesis

#### General description

The ^1^H (400 MHz) and ^13^C NMR (100 MHz) spectra were recorded on a Mercury 400 spectrometer from Varian (Palo Alto, CA). Chemical shifts are reported in ppm using either tetramethylsilane or the solvent peak as an internal standard. Data are reported as follows: chemical shift, multiplicity (s = singlet, brs = broad singlet, d = doublet, t = triplet, brt = broad triplet, q = quartet, m = multiplet), coupling constant, and integration. High-resolution mass spectra were obtained on a Bruker BioTOF II ESI. IR spectra were obtained on a Nicolet Continuμm Infrared Microscope FT-IR using KBr pellet. Medium pressure liquid chromatography (MPLC) was performed with Biotage SP-1 (Charlottesville, VA) using silica gel (EM Science, 230–400 mesh). HPLC was carried out on a 5-µm C18 column (analytical: 4.60×250 mm, HyperClone; semi-preparative: 21.2×250 mm, Gemini) from Phenomenex (Torrance, CA) eluting with linear gradient of 80% of solution A (1000 mL of H_2_O and 1 mL of TFA) to 100% of solution B (100 mL of H_2_O, 900 mL of MeCN and 1 mL of TFA) over 20 minutes at a flow rate of 1.0 mL/min (analytical) or over a specified amount of time at a flow rate of 10 mL/min (semi-preparative) with UV detection at 254 nm on a Beckman Coulter System Gold HPLC system (166P detector and 125P solvent module) from Beckman Coulter (Brea, CA). Benzotriazol-1-yloxytris(dimethylamino)phosphonium hexafluorophosphate, 1-hydroxy benzotriazole, *para*-toluene sulfonic acid hydrate, and *N*-methyl morpholine are abbreviated as BOP, HOBt, *p*-TSA hydrate, and NMM, respectively. KCN is highly toxic and must be handled with extreme care by trained personnel.

#### Synthesis of intermediate 14

2,2′-(6,6′-(Benzylazanediyl)bis(hexane-6,1-diyl))diisoindoline-1,3-dione (**13x**). To a stirred solution of **12x** (10.73 g, 30.8 mmol) in MeCN (250 mL) was added K_2_CO_3_ (7.74 g, 56.0 mmol) and benzylamine (1.50 g, 14.0 mmol). The resulting solution was refluxed for 36 hours. The reaction mixture was cooled to room temperature, filtered and the filtrate was concentrated *in vacuo*. MPLC purification of the residue (Hex∶EtOAc/70∶30) gave **13x** as a yellow viscous oil (6.7 g, 85%). ^1^H NMR (CDCl_3_) δ 7.83–7.77 (m, 4H), 7.71–7.65 (m, 4H), 7.29–7.23 (m, 4H), 7.20–7.14 (m, 1H), 3.63 (t, *J* = 7.2 Hz, 4H), 3.48 (s, 2H), 2.34 (t, *J* = 7.2 Hz, 4H), 1.68–1.58 (m, 4H), 1.48–1.36 (m, 4H), and 1.33–1.24 (m, 8H); ^13^C NMR (CDCl_3_) δ 168.65, 140.32, 134.04, 132.37, 129.04, 128.27, 126.83, 123.35, 58.74, 53.77, 38.24, 28.86, 27.21, and 27.01.

2,2′-(6,6′-Azanediylbis(hexane-6,1-diyl))diisooindoline-1,3-dione (**14x**). To a stirred solution of **13x** (1.00 g, 1.8 mmol) in MeOH∶EtOAc (1∶1, 20 mL each) was added 10% Pd-C (0.35 g). The resulting mixture was stirred at room temperature for 24 hours under a balloon of hydrogen. The reaction mixture was filtered through Celite and the filtrate was concentrated *in vacuo*. MPLC purification of the residue (Hex∶EtOAc/70∶30) gave **14x** as an amorphous white solid (0.82 g, 98%). ^1^H NMR (CD_3_OD) δ 7.84–7.78 (m, 8H), 3.67 (t, *J* = 7.2 Hz, 4H), 2.97 (t, *J* = 7.6 Hz, 4H), 1.74–1.62 (m, 8H), and 1.50–1.34 (m, 8H); ^13^C NMR (DMSO-*d_6_*) δ 168.65, 135.08, 132.25, 123.68, 47.27, 37.95, 28.47, 26.49, 26.28, and 26.20.

2,2′-(7,7′-(Benzylazanediyl)bis(heptane-7,1-diyl))diisoindoline-1,3-dione (**13y**). Intermediate **13y** was obtained as a yellow oil (6.29 g, 81%) from **12y** (9.40 g, 29.0 mmol), K_2_CO_3_ (7.60 g, 55.0 mmol) and benzylamine (1.48 g, 13.8 mmol) following the procedure described for the synthesis of **13x**. ^1^H NMR (CDCl_3_) δ 7.86–7.80 (m, 4H), 7.72–7.66 (m, 4H), 7.30–7.24 (m, 4H), 7.24–7.16 (m, 1H), 3.65 (t, *J* = 7.2 Hz, 4H), 3.50 (s, 2H), 2.35 (t, *J* = 7.2 Hz, 4H), 1.70–1.62 (m, 4H), 1.48–1.38 (m, 4H), and 1.38–1.18 (m, 12H); ^13^C NMR (CDCl_3_) δ 168.61, 140.42, 134.04, 132.37, 129.03, 128.24, 126.79, 123.33, 58.80, 53.91, 38.24, 29.33, 28.80, 27.48, 27.13, and 27.07.

2,2′-(7,7′-Azanediylbis(heptane-7,1-diyl))diisidioindoline-1,3-dione (**14y**). Intermediate **14y** was obtained as an as an amorphous white solid (1.05 g, 82%) from **13y** (1.50 g, 2.5 mmol) following the procedure described for the synthesis of **14x**. ^1^H NMR (CD_3_OD) δ 7.85–7.81 (m, 4H), 7.81–7.76 (m, 4H), 3.65 (t, *J* = 7.2 Hz, 4H), 2.56 (t, *J* = 7.6 Hz, 4H), 1.70–1.60 (m, 4H), 1.55–1.45 (m, 4H), and 1.45–1.30 (m, 12H); ^13^C NMR (DMSO-*d_6_*) δ 168.59, 135.03, 132.24, 123.65, 49.96, 38.03, 29.99, 29.21, 28.55, 27.37, and 26.93.

2,2′-(10,10′-(Benzylazanediyl)bis(decane-10,1-diyl))diisoindoline-1,3-dione (**13z**). Intermediate **13z** was obtained as a yellow viscous oil (5.67 g, 86%) from **12z** (7.5 g, 20.5 mmol), K_2_CO_3_ (5.36 g, 38.8 mmol) and benzylamine (1.04 g, 9.7 mmol) following the procedure described for the synthesis of **13x**. ^1^H NMR (CDCl_3_) δ 7.86–7.80 (m, 4H), 7.72–7.69 (m, 4H), 7.32–7.19 (m, 5H), 3.67 (t, *J* = 7.2 Hz, 4H), 3.52 (s, 2H), 2.36 (t, *J* = 7.2 Hz, 4H), 1.70–1.60 (m, 4H), 1.47–1.38 (m, 4H), and 1.38–1.18 (m, 24H).^ 13^C NMR (CDCl_3_) δ 168.62, 140.06 134.03, 132.36, 129.10, 128.27, 126.86, 123.33, 58.69, 53.89, 38.25, 29.76, 29.72, 29.66, 29.55, 29.39, 28.81, 27.61, 27.07, and 27.02.

2,2′-(10,10′-Azanediylbis(decane-10,1-diyl))diisoindoline-1,3-dione (**14z**). Intermediate **14z** was obtained as an as an amorphous white solid (602 mg, 55%) from **13z** (1.27 g, 1.87 mmol) following the procedure described for the synthesis of **14x**. ^1^H NMR (CD_3_OD) δ 7.86–7.81 (m, 4H), 7.81–7.76 (m, 4H), 3.65 (t, *J* = 7.2 Hz, 4H), 2.96 (t, *J* = 7.6 Hz, 4H), 1.72–1.60 (m, 8H), and 1.44–1.28 (m, 24H). 13C NMR (DMSO-*d6*) δ 168.61, 135.06, 132.23, 123.66, 47.28, 38.03, 29.44, 29.18, 28.54, 26.91, 26.66, and 26.04.

#### Synthesis of analog 8

*tert*-Butyl 5-bromo-4-methylthiophene-2-carboxylate (**16**). 4-Methylthiophene-2-carboxylic acid [48] (1.42 g, 10.00 mmol) was dissolved in MeCN (30 mL), followed by NBS (1.78 g, 10.00 mmol) at room temperature. After 16-hour stirring, solvent was removed *in vacuo*, the residue purified by MPLC (gradient Hex to EtOAc) to give 5-bromo-4-methylthiophene-2-carboxylic acid (2.19 g, 99%). ^1^H NMR (CDCl_3_) δ 9.60 (brs, 1H), 7.56 (s, 1H), and 2.22 (s, 3H); ^13^C NMR (CDCl_3_) δ 166.92, 139.17, 136.66, 131.62, 119.99, and 15.49. The resulting acid was converted to **16** (82%) following the literature procedure [49]. ^1^H NMR (CDCl_3_) δ 7.38 (s, 1H), 2.18 (s, 3H), and 1.55 (s, 9H); ^13^C NMR (CDCl_3_) δ 160.90, 138.42, 134.71, 134.35, 116.95, 82.33, 28.41, and 15.46.

*tert*-Butyl 5-bromo-4-formylthiophene-2-carboxylate (**17**). Compound **16** (0.80 g, 2.89 mmol) and a catalytic amount of AIBN were dissolved in benzene (10 mL), followed by NBS (0.51 g, 2.89 mmol) at room temperature. The resulting mixture was heated to reflux for 15 hours. Solvent was removed *in vacuo*, the residue purified by MPLC (gradient Hex to EtOAc) to give an inseparable mixture of the starting material/desired product/dibromination by-product at a ratio of 2∶7∶1 in total 75% yield. ^1^H NMR (CDCl_3_) δ 7.58 (s, 1H), 4.40 (s, 2H), 1.55 (s, 9H). The mixture (1.68 g) was subjected to the Hass-Bender reaction [50]. MPLC purification (gradient Hex to EtOAc) afforded **17** as a pale yellow solid (0.91 g, 66%). ^1^H NMR (CDCl_3_) δ 9.90 (s, 1H), 7.87 (s, 1H), and 1.56 (s, 9H).

(*E*)-*tert*-Butyl 5-bromo-4-(3-ethoxy-3-oxoprop-1-enyl)thiophene-2-carboxylate (**18**). Aldehyde **17** (0.91 g, 3.13 mmol) was dissolved in benzene (12 mL), followed by (ethoxycarbonylmethylene)triphenylphosphorane (2.18 g, 6.26 mmol), and then heated at 80°C for 19 hours. Solvent was evaporated, and MPLC purification of the residue (gradient Hex to EtOAc) afforded **18** as a viscous oil (1.13 g, quantitative). ^1^H NMR (CDCl_3_) δ 7.71 (s, 1H), 7.60 (d, *J* = 16.0 Hz, 1H), 6.34 (d, *J* = 16.0 Hz, 1H), 4.27 (q, *J* = 7.1 Hz, 2H), 1.56 (s, 9H), and 1.34 (t, *J* = 7.1 Hz, 3H).

(*E*)-*tert*-Butyl 4-(3-ethoxy-3-oxoprop-1-enyl)-5-(3-hydroxy-3,3-diphenylprop-1-ynyl)thiophene-2-carboxylate (**19**). To a stirred mixture of compound **18** (0.34 g, 0.94 mmol), 1,1-diphenylprop-2-yn-1-ol (0.39 g, 1.87 mmol), CuI (25 mg, 0.13 mmol), and PdCl_2_(PPh_3_)_2_ (46 mg, 0.07 mmol) in DMF (7 mL) was added Et_3_N (1.25 mL) at room temperature. After 17-hour stirring, H_2_O (5 mL) was added, extracted with EtOAc (3×10 mL), the extracts were combined, washed with brine, dried over MgSO_4_, filtered, concentrated, and purified by MPLC (gradient Hex to EtOAc) to give **19** in a quantitative yield. ^1^H NMR (CDCl_3_) δ 7.73 (s and d, for d, *J* = 15.9 Hz, 1H each), 7.64 (d, *J* = 7.6 Hz, 4H), 7.38–7.34 (m, 4H), 7.29 (t, *J* = 7.2 Hz, 2H), 6.37 (d, *J* = 15.9 Hz), 4.21 (q, *J* = 7.2 Hz, 2H), 1.56 (s, 9H), and 1.28 (t, *J* = 7.2 Hz, 3H); ^13^C NMR (CDCl_3_) δ 166.96, 160.51, 144.42, 140.75, 136.45, 135.92, 130.05, 128.84, 128.70, 128.65, 128.57, 128.22, 126.27, 120.51, 83.16, 78.52, 75.33, 60.92, 28.36, 21.29, and 14.52.

*tert*-Butyl 4-(3-ethoxy-3-oxopropyl)-5-(3-hydroxy-3,3-diphenylpropyl)thiophene-2-carboxylate (**20**). To a stirred solution of **19** (0.46 g, 0.94 mmol) in MeOH (15 mL) Raney-Ni (1.0 mL as MeOH slurry) was added, and the stirring was continued under a balloon of hydrogen for 17 hours. The metal catalyst was removed with a magnet, the catalyst was washed with MeOH. The washings and the aliquot were combined, concentrated *in vacuo*, purified by MPLC (gradient Hex to EtOAc) to give **20** as an oil (0.42 g, 91%). ^1^H NMR (CDCl_3_) δ 7.45 (d, *J* = 7.8 Hz, 4H), 7.41 (s, 1H), 7.34–7.30 (m, 4H), 7.23 (t, *J* = 7.2 Hz, 2H), 4.09 (q, *J* = 7.2 Hz, 2H), 2.77–2.70 (m, 4H), 2.62–2.58 (m, 3H), 2.47 (t, *J* = 7.5 Hz, 2H), 1.54 (s, 9H), and 1.21 (t, *J* = 7.2 Hz, 3H); ^13^C NMR (CDCl_3_) δ 172.93, 161.79, 147.52, 146.65, 136.99, 134.24, 131.66, 128.56, 127.30, 126.15, 81.65, 78.03, 60.82, 44.10, 35.05, 28.47, 23.36, 23.08, and 14.41.

5-(3,3-Diphenylallyl)-4-(3-ethoxy-3-oxopropyl)thiophene-2-carboxylic acid (**21**). Intermediate **20** (0.38 g, 0.76 mmol) and *p*-TSA hydrate (0.15 g, 0.76 mmol) were dissolved in benzene (10 mL) at room temperature. The resulting mixture was heated at 70–75°C for 40 minutes. The reaction mixture was washed with water (10 mL), dried over MgSO_4_, concentrated, and the residue was purified by MPLC (gradient Hex to EtOAc) to give **21** as an oily residue (0.23 g, 65%). ^1^H NMR (CDCl_3_) δ 10.80 (brs, 1H), 7.65 (s, 1H), 7.42–7.35 (m, 4H), 7.29–7.24 (m, 6H), 6.21 (t, *J* = 7.4 Hz, 1H), 4.11 (q, *J* = 7.2 Hz, 2H), 3.61 (d, *J* = 7.4 Hz, 2H), 2.78 (t, *J* = 7.6 Hz, 2H), 2.51 (t, *J* = 7.6 Hz, 2H), and 1.22 (t, *J* = 7.2 Hz, 3H); ^13^C NMR (CDCl_3_) δ 172.72, 167.85, 149.21, 144.52, 141.95, 139.35, 137.59, 136.58, 129.94, 128.72, 128.46, 127.77, 127.68, 124.99, 60.88, 34.83, 29.21, 23.52, and 14.43.

Ethyl 3-(5-(bis(10-(1,3-dioxoisoindolin-2-yl)decyl)carbamoyl)-2-(3,3-diphenylallyl)thiophen-3-yl)propanoate (**22**). To a stirred mixture of **21** (109 mg, 0.26 mmol), **14z** (152 mg, 0.26 mmol), BOP (115 mg, 0.26 mmol), and HOBt (35 mg, 0.26 mmol) in dry DMF (3 mL) was added NMM (34.2 µL, 0.31 mmol) at room temperature. After 13 hours stirring, H_2_O (5 mL) was added, extracted with EtOAc (3×5 mL). The extracts were combined, washed with brine, dried over MgSO_4_, filtered, concentrated, and then purified by MPLC (gradient Hex to EtOAc) to give **22** as an oil (187 mg, 73%). ^1^H NMR (CDCl_3_) δ 7.80–7.78 (m, 4H), 7.67–7.65 (m, 4H), 7.37–7.29 (m, 3H), 7.24–7.17 (m, 7H), 7.05 (s, 1H), 6.15 (t, *J* = 7.4 Hz, 1H), 4.06 (q, *J* = 7.2 Hz, 2H), 3.63 (t, *J* = 7.1 Hz, 4H), 3.52 (d, *J* = 7.5 Hz, 2H), 3.40–3.39 (m, 4H), 2.71 (t, *J* = 7.5 Hz, 2H), 2.44 (t, *J* = 7.5 Hz, 2H), 1.62–1.59 (m, 8H), and 1.23–1.51 (m, 24H); ^13^C NMR (CDCl_3_) δ 172.72, 168.62, 164.20, 143.60, 142.11, 142.05, 139.49, 135.75, 134.77, 134.86, 134.06, 132.36, 130.48, 130.00, 128.60, 128.36, 127.61, 127.55, 125.96, 123.33, 60.67, 38.22, 35.07, 29.69, 29.61, 29.57, 29.34, 28.80, 28.58, 27.03, 23.57, and 14.44.

Ethyl 3-(5-(bis(10-(1,3-dioxoisoindolin-2-yl)decyl)carbamoyl)-2-(3,3-diphenylpropyl)thiophen-3-yl)propanoate (**23**). To a stirred solution of compound **15** (187 mg, 0.19 mmol) in EtOAc (5 mL) was added 10% Pd-C (40 mg). The resulting suspension was stirred under a balloon of hydrogen for 20 hours at room temperature. The catalyst was removed by filtration through Celite, and concentration of the filtrate gave **23** (171 mg, 91%). ^1^H NMR (CDCl_3_) δ 7.84–7.81 (m, 4H), 7.70–7.68 (m, 4H), 7.30–7.23 (m, 8H), 7.18–7.15 (m, 2H), 7.04 (s, 1H), 4.07 (q, *J* = 7.2 Hz, 2H), 3.98 (t, *J* = 7.8 Hz, 1H), 3.66 (t, *J* = 7.2 Hz, 4H), 3.44–3.40 (m, 4H), 2.71–2.63 (m, 4H), 2.44–2.35 (m, 4H), 1.65–1.61 (m, 8H), and 1.54–1.19 (m, 27H); ^13^C NMR (CDCl_3_) δ 172.80, 168.71, 164.26, 144.43, 142.95, 135.92, 134.33, 134.08, 132.38, 130.41, 128.81, 128.62, 128.04, 126.58, 123.38, 60.69, 50.68, 38.27, 37.49, 35.17, 29.73, 29.64, 29.60, 29.37, 28.83, 27.07, 26.33, 23.47, and 14.46.

*N,N*-Bis(10-aminodecyl)-5-(3,3-diphenylpropyl)-4-(3-(hydroxyamino)-3-oxopropyl)thiophene-2-carboxamide (**8**). A stirred solution of **23** (171 mg, 0.17 mmol) in MeOH∶THF (2 mL each) containing 50% aqueous NH_2_OH (1 mL) and a catalytic amount (two crystals) of KCN was heated at 65°C for 24 hours. The resulting solution was filtered through Celite, concentrated, re-dissolved in MeOH, and purified by HPLC (eluting time: 20 minutes) to give of **8•2TFA** (23 mg, 14%). Both semi-preparative and analytical HPLC retention times of **8•2TFA** are 14.83 minutes (see Figure S7 for chromatograms of **8•2TFA** before and after the HPLC purification). ^1^H NMR (CD_3_OD) δ 7.28–7.27 (m, 8H), 7.20–7.15 (m, 2H), 7.11 (s, 1H), 4.00 (t, *J* = 7.8 Hz, 1H), 3.50–3.40 (brs, 4H), 2.88 (t, *J* = 7.2 Hz, 4H), 2.74 (t, *J* = 7.6 Hz, 2H), 2.67 (t, *J* = 7.2 Hz, 2H), 2.39 (q, *J* = 7.6 Hz, 2H), 2.23 (t, *J* = 7.2 Hz, 2H), 1.70–1.52, (m, 8H), and 1.40–1.20 (m, 24H); ^13^C NMR (CD_3_OD) δ 170.24, 165.32, 160.34 (q), 144.59, 143.32, 136.41, 133.15, 130.77, 128.44, 128.24, 128.18, 127.73, 126.24, 125.90, 116.58 (q), 50.59, 39.54, 37.44, 33.48, 29.32, 29.20, 29.15, 28.98, 27.36, 26.58, 26.23, 25.82, and 23.61; IR cm^−1^ 3060, 3028, 2921, 2852, 1679, 1589, 1205, and 1136; HRMS-ESI calculated for C_43_H_66_N_4_O_3_S [M+H^+^] 719.4928, found 719.4956.

#### Synthesis of analog 9

Ethyl 2-(2-Bromothiophen-3-yl)acetate (**25**). To a solution of **24** (20.30 g, 119.25 mmol) in THF (150 mL) was added NBS (21.23 g, 119.25 mmol) over a period of 5 hours at 0°C, and then the mixture was warmed up to room temperature, stirring continued for 24 hours. The solvent was removed *in vacuo*, and the residue was dissolved in EtOAc (150 mL), washed with brine (4×30 mL). The organic layer was dried over MgSO_4_, filtered, and concentrated *in vacuo*. MPLC purification (Hex∶EtOAc/5∶1) of the residue gave **25** as a colorless oil (24.00 g, 81%). ^1^H NMR (CDCl_3_) δ 7.23 (d, *J* = 5.4 Hz, 1H), 6.93 (d, *J* = 5.4 Hz, 1H), 4.17 (q, *J* = 7.2 Hz, 2H), 3.61 (s, 2H), and 1.27 (t, *J* = 7.2 Hz, 3H); ^13^C NMR (CDCl_3_) δ 170.37, 133.84, 128.90, 125.93, 111.78, 61.32, 35.30, and 14.43.

Ethyl 2-(2-bromo-5-(chlorocarbonyl)thiophen-3-yl)acetate (**26**). A solution of oxalyl chloride (2.00 g, 16 mmol) in CH_2_Cl_2_ (3 mL) was added dropwise into a suspension of AlCl_3_ (1.064 g, 8 mmol) in CH_2_Cl_2_ (6 mL) at 0°C. After the mixture was stirred for 1 hour, a solution of **25** (1.00 g, 4 mmol) in CH_2_Cl_2_ (2 mL) was added into the mixture during a period of 1 hour at 0°C. The mixture was allowed to warm up to room temperature and stirred for additional 3 hours. The reaction mixture was poured into ice cold H_2_O (20 mL) and extracted with Et_2_O (100 mL). The organic layer was washed with brine (3×15 mL), dried over MgSO_4_, and concentrated *in vacuo*. MPLC purification (Hex∶EtOAc/20∶1) of the residue gave **26** as a colorless oil (682 mg, 54%). ^1^H NMR (CDCl_3_) δ 7.82 (s, 1H), 4.19 (q, *J* = 7.2 Hz, 2H), 3.64 (s, 2H), and 1.28 (t, *J* = 7.2 Hz, 3H); ^13^C NMR (CDCl_3_) δ 169.30, 159.01, 139.01, 136.64, 136.46, 126.06, 61.79, 35.18, and 14.40.

4-Methoxybenzyl 5-bromo-4-(2-ethoxy-2-oxoethyl)thiophene-2-carboxylate (**27**). To a solution of 4-methoxybenzyl alcohol (220 µL, 1.75 mmol) and Et_3_N (244 µL, 1.75 mmol) in CH_2_Cl_2_ (12 mL) was added dropwise a solution of **26** (367 mg, 1.17 mmol) in CH_2_Cl_2_ (3 mL) at room temperature. After the mixture was stirred for 1 hour at room temperature, it was poured into H_2_O (20 mL) and extracted with Et_2_O (50 mL). The organic layer was washed with brine (2×10 mL), dried over MgSO_4_, and concentrated *in vacuo*. MPLC purification (Hex∶EtOAc/8∶1) of the residue gave **27** as a white solid (400 mg, 82%). ^1^H NMR (CDCl_3_) δ 7.61 (s, 1H), 7.34 (d, *J* = 8.4 Hz, 4H), 6.90 (d, *J* = 8.4 Hz, 2H), 5.23 (s, 2H), 4.16 (q, *J* = 7.2 Hz, 2H), 3.80 (s, 3H), 3.59 (s, 2H), and 1.26 (t, *J* = 7.2 Hz, 3H); ^13^C NMR (CDCl_3_) δ 169.76, 161.30, 159.99, 135.14, 134.73, 133.55, 130.45, 127.80, 119.89, 114.22, 67.14, 61.56, 55.53, 35.28, and 14.40.

4-Methoxybenzyl 4-(2-ethoxy-2-oxoethyl)-5-(3-hydroxy-3,3-diphenylprop-1-ynyl)thiophene-2-carboxylate (**28**). CuI (7 mg, 0.037 mmol) was added into a mixture of **27** (214 mg, 0.51 mmol), PdCl_2_(PPh_3_)_2_ (20 mg, 0.028 mmol), Et_3_N (1.046 g, 10.36 mmol) and 1,1-diphenylprop-2-yn-1-ol (237 mg, 1.14 mmol) in anhydrous DMF (4 mL). The mixture was stirred at room temperature for 2 days. The mixture was poured into H_2_O and extracted with Et_2_O (100 mL). The organic layer was washed with brine (4×20 mL), dried over MgSO_4_, and concentrated *in vacuo*. MPLC purification (Hex∶EtOAc/6∶1) of the residue gave **28** as a brown oil (206 mg, 74%).^1^H NMR (CDCl_3_) δ 7.67 (s, 1H), 7.62 (d, *J* = 7.6 Hz, 4H), 7.38−7.25 (m, 8H), 6.90 (d, *J* = 8.8 Hz, 2H), 5.24 (s, 2H), 4.10 (q, *J* = 7.0 Hz, 2H), 3.81 (s, 3H), 3.67 (s, 2H), and 1.20 (t, *J* = 7.2 Hz, 3H); ^13^C NMR (CDCl_3_) δ 170.18, 161.56, 159.98, 144.60, 139.49, 134.65, 133.54, 130.46, 128.64, 128.19, 127.83, 126.73, 126.24, 114.23, 101.51, 78.92, 75.28, 67.21, 61.54, 55.54, 35.33, and 14.37.

4-(2-Ethoxy-2-oxoethyl)-5-(3-hydroxy-3,3-diphenylpropyl)thiophene-2-carboxylic acid (**29**). To a solution of **28** (76 mg, 0.14 mmol) in dry MeOH (4 mL) was added 10% Pd-C (38 mg). The mixture was stirred under a balloon of hydrogen for 2 days at room temperature and filtered through Celite. Concentration of the filtrate *in vacuo* gave **29** as a colorless oil (50 mg, 83%). ^1^H NMR (CDCl_3_) δ 7.66 (s, 1H), 7.44 (d, *J* = 7.6 Hz, 4H), 7.33 (t, *J* = 7.6 Hz, 4H), 7.28−7.22 (m, 2H), 4.11 (q, *J* = 7.2 Hz, 2H), 3.44 (s, 2H), 2.82−2.75 (m, 2H), 2.67−2.60 (m, 2H), and 1.22 (t, *J* = 7.2 Hz, 3H); ^13^C NMR (CDCl_3_) δ 170.97, 167.28, 151.87, 146.49, 137.33, 131.03, 128.88, 128.60, 128.01, 127.37, 126.71, 126.14, 78.05, 61.50, 43.73, 34.09, 29.95, 23.52, and 14.37.

5-(3,3-Diphenylallyl)-4-(2-ethoxy-2-oxoethyl)thiophene-2-carboxylic acid (**30**). To a solution of **29** (22 mg, 0.052 mmol) in benzene (6 mL) was added *p*-TSA hydrate (9 mg, 0.052 mmol). After the mixture was stirred at 70°C for 1 hour, it was poured into H_2_O (20 mL) and extracted with Et_2_O (50 mL). The organic layer was washed with brine (2×15 mL), dried over MgSO_4_, and concentrated *in vacuo* to give **30** as a colorless oil (22 mg, quantitative yield).^ 1^H NMR (CDCl_3_) δ 7.71 (s, 1H), 7.44−7.20 (m, 10H), 6.20 (t, *J* = 7.4 Hz, 1H), 4.09 (q, *J* = 7.2 Hz, 2H), 3.60 (d, *J* = 7.2 Hz, 2H), 3.46 (s, 2H), and 1.19 (t, *J* = 7.2 Hz, 3H); ^13^C NMR (CDCl_3_) δ 170.63, 167.35, 150.56, 144.62, 141.88, 139.31, 137.52, 131.02, 129.93, 128.73, 128.44, 127.80, 127.68, 124.68, 104.60, 61.42, 34.13, 29.40, and 14.35.

5-(3,3-Diphenylpropyl)-4-(2-ethoxy-2-oxoethyl)thiophene-2-carboxylic acid (**31**). To a solution of **30** (22 mg, 0.054 mmol) in dry MeOH (5 mL) and EtOAc (1 mL) was added 10% Pd-C (4 mg). The mixture was stirred under a balloon of hydrogen for 12 hours at room temperature and filtered through Celite. Concentration of the filtrate *in vacuo* gave **31** as a colorless oil (22 mg, quantitative yield).^ 1^H NMR (CDCl_3_) δ 7.69 (s, 1H), 7.32−7.16 (m, 10H), 4.10 (q, *J* = 7.2 Hz, 2H), 3.98 (t, *J* = 8.0 Hz, 1H), 3.37 (s, 2H), 2.75 (t, *J* = 7.8 Hz, 2H), 2.43 (q, *J* = 8.0 Hz, 2H), and 1.21 (t, *J* = 7.2 Hz, 3H); ^13^C NMR (CDCl_3_) δ 170.69, 167.27, 151.15, 144.20, 137.27, 131.21, 129.05, 128.88, 128.02, 126.72, 61.39, 50.75, 37.13, 34.05, 27.20, and 14.37.

Ethyl 2-(5-(bis(10-(1,3-dioxoisoindolin-2-yl)decyl)carbamoyl)-2-(3,3-diphenylpropyl)thiophen-3-yl)acetate (**32**). A mixture of **31** (22 mg, 0.054 mmol), BOP (36 mg, 0.081 mmol), NMM (8 mg, 0.081 mmol), HOBt (11 mg, 0.081 mmol), and **14z** (47 mg, 0.081 mmol) in anhydrous DMF (4 mL) was stirred at room temperature under N_2_ for ∼24 hours. When the reaction was over by TLC monitoring, the mixture was poured into H_2_O (10 mL) and then extracted with Et_2_O (70 mL). The organic layer was washed with brine (3×10 mL), dried over MgSO_4_, and concentrated *in vacuo*. MPLC purification (Hex/EtOAc 4∶1) of the residue gave **32** as a yellow amorphous solid (32 mg, 74% based on recovery of starting material). ^1^H NMR (CDCl_3_) δ 7.86−7.80 (m, 4H), 7.73−7.68 (m, 4H), 7.31−7.15 (m, 10H), 7.13 (s, 1H), 4.08 (q, *J* = 7.2 Hz, 2H), 3.98 (t, *J* = 8.0 Hz, 1H), 3.67 (t, *J* = 7.4 Hz, 4H), 3.44 (brt, 4H), 3.33 (s, 2H), 2.70 (t, *J* = 7.6 Hz, 2H), 2.40 (q, *J* = 7.6 Hz, 2H), 1.70−1.57 (m, 8H), 1.36−1.23 (m, 24H), and 1.20 (t, *J* = 7.2 Hz, 3H); ^13^C NMR (CDCl_3_) δ 170.91, 168.70, 164.02, 144.60, 144.40, 134.69, 134.08, 132.39, 130.87, 129.36, 128.80, 128.04, 126.59, 123.38, 61.15, 50.66, 38.27, 37.32, 34.18, 29.72, 29.63, 29.58, 29.37, 28.82, 27.06, 26.54, and 14.40.

*N*,*N*-Bis(10-aminodecyl)-5-(3,3-diphenylpropyl)-4-(2-(hydroxyamino)-2-oxoethyl)thiophene-2-carboxamide (**9**). To a stirred solution of **32** (18 mg, 0.018 mmol) in THF/MeOH (1.5 mL/1.5 mL), 1 mL of 50% aqueous NH_2_OH was added, followed by catalytic amount (two crystals) of KCN. The resulting mixture was stirred for 23 hours at room temperature, and then filtered through a short Celite column. HPLC purification (eluting time: 30 minutes) of the filtrate gave **9•2TFA** as a white amorphous solid (18 mg, quantitative yield). The semi-preparative and analytical HPLC retention times of **9•2TFA** are 17.70 and 15.07 minutes, respectively (see Figure S8 for chromatograms of **9•2TFA** before and after the HPLC purification). ^1^H NMR (DMSO-*d*_6_) δ 10.62 (s, 1H), 7.69 (brs, 7H), 7.35−7.10 (m, 11H), 3.98 (t, *J* = 7.8 Hz, 1H), 3.37 (brs, 4H), 3.10 (s, 2H), 2.72 (m, 4H), 2.64 (t, *J* = 7.6 Hz, 2H), 2.33 (q, *J* = 7.2 Hz, 2H), 1.60−1.40 (m, 8H), 1.30−1.15 (m, 24H); ^13^C NMR (DMSO-*d*_6_) δ 167.04, 163.29, 158.66 (q), 145.18, 143.86, 134.23, 132.01, 131.52, 129.21, 128.26, 126.91, 50.68, 39.50, 39.40, 37.26, 32.69, 29.57, 29.47, 29.44, 29.19, 27.68, 27.62, 26.89, and 26.44; IR cm^−1^ 2934, 2852, 1683, 1589, 1205, and 1136; HRMS-ESI calculated for C_42_H_64_N_4_O_3_S [M+H^+^] 705.4772, found 705.4761.

#### Synthesis of analog 10b

Methyl 4-formyl-5-iodo-1*H*-pyrrole-2-carboxylate (**34**). To a stirred solution of **33** (3.40 g, 22.2 mmol) in CHCl_3_ (20 mL) at 0°C was added CF_3_CO_2_Ag (5.40 g, 24.4 mmol) followed by adding I_2_ in three portions each with 2.07 g (8.13 mmol) of the material. The resulting mixture was stirred for 16 hours in dark at room temperature. The reaction mixture was filtered through Celite and the filtrate was washed with saturated aqueous solution of Na_2_S_2_O_3_. The organic layer was dried over anhydrous MgSO_4_, filtered and concentrated *in vacuo*. MPLC purification (CH_2_Cl_2_∶Et_2_O/95∶5) of the residue gave **34** as a white solid (1.90 g, 29%). Along with **34**, two minor compounds methyl 4-formyl-5-iodo-1*H*-pyrrole-2-carboxylate, methyl 4-formyl-3,5-diiodo-1*H*-pyrrole-2-carboxylate and the starting material **33** were also isolated. ^1^H NMR (DMSO-*d_6_*) δ 13.31 (brs, 1H), 9.52 (s, 1H), 7.04 (s, 1H), and 3.76 (s, 3H); ^13^C NMR (DMSO-*d*_6_) δ 187.13, 160.36, 128.78, 127.79, 115.58, 87.40, and 52.42.

Methyl 1-benzyl-4-formyl-5-iodo-1*H*-pyrrole-2-carboxylate (**35x**). To a stirred solution of **34** (0.72 g, 2.56 mmol) in acetone (20 mL) was added K_2_CO_3_ (0.71 g, 5.12 mmol). The resulting mixture was stirred for 30 minutes at room temperature. Benzyl bromide (0.46 mL, 3.84 mmol) was added dropwise to the reaction mixture and was refluxed for 16 hours. The reaction mixture was cooled to room temperature, concentrated and partitioned twice between ethyl acetate (10 mL) and water (10 mL). The aqueous layer was washed with EtOAc (10 mL). The combined organic layer was dried over anhydrous MgSO_4_, filtered and then concentrated *in vacuo*. MPLC purification (Hex∶EtOAc/80∶20) of the residue gave **35x** as a white solid (0.85 g, 90%). ^1^H NMR (CDCl_3_) δ 9.66 (s, 1H), 7.50 (s, 1H), 7.25–7.18 (m, 3H), 6.98 (d, *J* = 7.6 Hz, 2H), 5.75 (s, 2H), and 3.72 (s, 3H); ^13^C NMR (CDCl_3_) δ 186.98, 160.33, 136.43, 128.97, 128.22, 127.84, 127.10, 126.59, 119.32, 92.81, 52.81, and 52.11.

Methyl 1-benzyl-4-formyl-5-(3-hydroxy-3,3-diphenylprop-1-ynyl)-1*H*-pyrrole-2-carboxylate (**36x**). A solution of **35x** (0.47 g, 1.26 mmol), 1,1-diphenylprop-2-yn-1-ol (0.66 g, 3.15 mmol), CuI (0.048 g, 0.25 mmol), Pd(PPh_3_)_2_Cl_2_ (0.18 g, 0.3 mmol), PPh_3_ (0.066 g, 0.3 mmol), and Et_3_N (4 mL) in anhydrous DMF (4 mL) was stirred for 16 hours at 70°C under N_2_ atmosphere. The reaction mixture was partitioned between water and EtOAc. The combined organic layer was dried over MgSO_4_, filtered and concentrated *in vacuo*. MPLC purification (Hex∶EtOAc/80∶20) of the residue gave **36x** as a pale yellow solid (0.49 g, 87%). ^1^H NMR (CDCl_3_) δ 9.88 (s, 1H), 7.52–7.50 (m, 4H), 7.36 (s, 1H), 7.30–7.24 (m, 9H), 7.08–7.02 (m, 2H), 5.69 (s, 2H), 3.80 (s, 3H), and 3.65 (s, 1H); ^13^C NMR (CDCl_3_) δ 185.16, 160.72, 144.07, 136.84, 128.91, 128.72, 128.33, 127.91, 127.79, 127.05, 126.23, 124.96, 116.97, 103.35, 75.37, 75.27, 52.15, and 50.38. ^1^H-^13^C HMBC (CDCl_3_): there is a correlation between the pyrrole proton and the carbonyl carbon of the methyl ester, and there is no correlation between the pyrrole proton and the benzylic carbon.

1-Benzyl-4-formyl-5-(3-hydroxy-3,3-diphenylprop-1-ynyl)-1*H*-pyrrole-2-carboxylic acid (**37x**). To a solution of **36x** (0.49 g, 1.09 mmol) in MeOH (20 mL) and water (6 mL) was added KOH (0.18 g, 3.27 mmol) and stirred at 60°C for 2 hours. The solvent was removed *in vacuo*. The residue obtained was acidified with 2 N HCl to pH 2–3 and extracted with EtOAc (3×15 mL). The combined organic layer was dried over MgSO_4_, filtered and then concentrated *in vacuo* to give **37x** as a light brown solid. The crude product was subjected to the next reaction without further purification. ^1^H NMR (CDCl_3_) δ 9.82 (s, 1H), 7.52–7.50 (m, 4H), 7.45 (s, 1H), 7.30–7.18 (m, 9H), 7.06–6.99 (m, 2H), and 5.64 (s, 2H).

1-Benzyl-*N*,*N*-bis(6-(1,3-dioxoisoindolin-2-yl)hexyl)-4-formyl-5-(3-hydroxy-3,3-diphenylprop-1-ynyl))-1*H*-pyrrole-2-carboxamide (**38x**). A solution of **37x** (0.35 g, 0.81 mmol), **14x** (0.46 g, 0.97 mmol), BOP (0.73 g, 1.61 mmol), HOBt (0.22 g, 1.61 mmol) in anhydrous DMF (3 mL) and NMM (2 mL) was stirred at room temperature for 16 hours. The reaction mixture was partitioned between water and CH_2_Cl_2_ (20 mL). The organic layer was dried over MgSO_4_, filtered and concentrated *in vacuo*. MPLC purification (Hex∶EtOAc/40∶60) of the residue gave **38x** as a pale yellow oil. (0.30 g, 41%). ^1^H NMR (CDCl_3_) δ 9.86 (s, 1H), 7.82–7.75 (m, 4H), 7.70–7.62 (m, 4H), 7.56–7.54 (m, 4H), 7.27–7.21 (m, 6H), 7.15–7.14 (m, 3H), 7.02–6.98 (m, 2H), 6.58 (s, 1H), 5.34 (s, 2H), 3.68–3.52 (m, 4H), 3.28–3.18 (m, 2H), 3.04–2.94 (m, 2H), 1.68–1.59 (m, 2H), 1.58–1.46 (m, 2H), 1.42–1.27 (m, 6H), 1.18–1.04 (m, 4H), and 1.02–0.94 (m, 2H); ^13^C NMR (CDCl_3_) δ 185.34, 168.68, 162.62, 144.57, 136.84, 134.15, 132.26, 129.37, 128.87, 128.61, 128.11, 127.79, 127.29, 126.20, 125.18, 123.42, 109.14, 102.74, 75.31, 75.10, 49.57, 45.39, 37.96, 28.57, 27.13, 26.73, 26.48, and 26.28.

(*E*)-Ethyl 3-(1-benzyl-5-bis(6-(1,3-dioxoisoindolin-2-yl)hexyl)carbamoyl)-2-(3-hydroxy-3,3-diphenylprop-1-ynyl))-1*H*-pyrrol-3-yl)acrylate (**39x**). To a stirred solution of **38x** (0.33 g, 0.37 mmol) in benzene (20 mL) was added (ethoxycarbonylmethylene)triphenylphosphorane (0.35 g, 0.92 mmol). The resulting mixture was stirred under reflux for 16 hours. The reaction mixture was cooled to room temperature and the solvent was removed *in vacuo*. MPLC purification (Hex∶EtOAc/50∶50) of the residue gave **39x** as a pale yellow oil (0.30 g, 85%). ^1^H NMR (CDCl_3_) δ 7.86–7.79 (m, 4H), 7.72–7.65 (m, 5H), 7.57–7.55 (m, 4H), 7.31–7.22 (m, 6H), 7.20–7.15 (m, 3H), 7.06–6.98 (m, 2H), 6.42 (s, 1H), 6.25 (d, *J* = 15.6 Hz, 1H), 5.33 (s, 2H), 4.18 (q, *J* = 7.2 Hz, 2H), 3.70–3.58 (m, 4H), 3.44 (s, 1H), 3.32–3.02 (m, 4H), 1.70–1.50 (m, 4H), and 1.49–1.12 (m, 15H); ^13^C NMR (CDCl_3_) δ 168.68, 167.65, 163.07, 144.63, 137.59, 136.76, 134.14, 132.32, 129.43, 128.98, 128.79, 128.66, 128.61, 128.06, 127.86, 127.77, 127.57, 126.24, 123.97, 123.42, 119.74, 115.04, 108.54, 102.26, 77.18, 75.34, 64.61, 60.36, 49.60, 38.00, 30.86, 28.64, 26.75, and 14.44.

Ethyl 3-(1-benzyl-5-(bis(6-(1,3-dioxoisoindolin-2-yl)hexyl)carbamoyl)-2-(3-hydroxy-3,3-diphenylpropyl)-1*H*-pyrrol-3-yl)propanoate (**40x**). To a stirred solution of 3**9x** (0.15 g, 0.16 mmol) in EtOAc (20 mL) was added 10% dry Pd-C (0.015 g). The resulting mixture was stirred at room temperature for 48 hours under a balloon of hydrogen. The reaction mixture was filtered through Celite and the filtrate was concentrated to give **40x** as a colorless oil. The crude product was subjected to the next reaction without further purification. ^1^H NMR of the crude product is as follows. ^1^H NMR (CDCl_3_) δ 7.77–7.75 (m, 4H), 7.64–7.62 (m, 4H), 7.27–7.06 (m, 13H), 6.71 (d, *J* = 7.2 Hz, 2H), 6.05 (s, 1H), 5.09 (s, 2H), 3.98 (q, *J* = 6.8 Hz, 2H), 3.57 (t, *J* = 7.2 Hz, 4H), 3.19 (t, *J* = 6.8 Hz, 4H), 2.56 (t, *J* = 7.6 Hz, 2H), 2.41–2.37 (m, 4H), 2.26–2.24 (m, 2H), 1.56–1.53 (m, 4H), and 1.30–1.11 (m, 15H).

*N*,*N*-Bis(6-aminohexyl)-1-benzyl-5-(3-(hydroxy-3,3-diphenylpropyl)-4-(3-hydroxyamino)-3-oxopropyl))-1*H*-pyrrole-2-carboxamide (**10b**). To a stirred solution of **40x** (0.15 g, 0.16 mmol) in MeOH∶THF (3 mL each) was added 50% aqueous hydroxylamine (4 mL) followed by KCN (0.04 g, 0.62 mmol). The resulting mixture was stirred at 35°C for 24 hours. After the solvent was removed *in vacuo*, the residue was subjected to HPLC purification (eluting time: 45 minutes). The eluent from HPLC was concentrated *in vacuo* at room temperature to remove the organic solvents. The resulting aqueous solution was lyophilized to give **10b•2TFA** as a white sticky solid (0.032 g, 22%). The semi-preparative and analytical HPLC retention times of **10b•2TFA** are 18.27 and 11.50 minutes, respectively (see Figure S9 for chromatograms of **10b•2TFA** before and after the HPLC purification). ^1^H NMR (DMSO-*d_6_*) δ 10.40 (brs, 1H), 7.70 (brs, 6H), 7.42–7.40 (m, 4H), 7.26–7.12 (m, 9H), 6.68 (d, *J* = 7.2 Hz, 2H), 6.10 (s, 1H), 5.15 (s, 2H), 3.26–3.18 (m, 4H), 3.14 (s, 1H), 2.74–2.68 (m, 4H), 2.45 (overlapped with DMSO-*d_6_*, 2H), 2.34–2.28 (m, 4H), 2.17 (t, *J* = 7.2 Hz, 2H), 1.49–1.41 (m, 4H), 1.36–1.25 (m, 4H), 1.24–1.16 (m, 4H), and 1.14–1.02 (m, 4H); ^13^C NMR (DMSO-*d_6_*) δ 169.44, 164.17, 158.86 (q), 148.59, 140.07, 133.78, 128.93, 128.54, 127.44, 126.86, 126.48, 126.28, 124.81, 118.30, 115.50 (q), 111.50, 76.72, 47.24, 42.30, 39.40, 34.50, 27.60, 26.41, 26.20, 21.75, and 19.05; IR cm^−1^ 3407, 3089, 3060, 3032, 2946, 2860, 1683, 1597, 1495, 1209, and 1140; HRMS-ESI calculated for C_42_H_57_N_5_O_4_ [M+H^+^] 696.4483, found 696.4510.

#### Synthesis of analog 11

Methyl 4-formyl-5-iodo-1-methyl-1*H*-pyrrole-2-carboxylate (**35y**). To a stirred solution of **34** (0.30 g, 1.08 mmol) in acetone (10 mL) was added K_2_CO_3_ (0.30 g, 2.15 mmol). The resulting mixture was stirred for 30 minutes at room temperature. Methyl iodide (0.23 g, 1.62 mmol) was added drop wise to the reaction mixture and was refluxed for 16 hours. The reaction mixture was cooled to room temperature, concentrated and partitioned between ethyl acetate (10 mL) and water (10 mL). The aqueous layer was washed with EtOAc (2×10 mL). The combined organic layer was dried over anhydrous MgSO_4_, filtered and concentrated *in vacuo*. MPLC purification (Hex∶EtOAc/80∶20) of the residue gave **35y** as a white solid (0.20 g, 63%). ^1^H NMR (CDCl_3_) δ 9.60 (s, 1H), 7.39 (s, 1H), 3.98 (s, 3H), and 3.80 (s, 3H); ^13^C NMR (CDCl_3_) δ 186.84, 160.71, 128.32, 126.61, 118.76, 92.60, 52.05, and 38.00.

Methyl 4-formyl-5-(3-hydroxy-3,3-diphenylprop-1-ynyl)-1-methyl-1*H*-pyrrole-2-carboxylate (**36y**). A solution of **35y** (0.20 g, 0.68 mmol), 1,1-diphenylprop-2-yn-1-ol (0.36 g, 1.7 mmol), CuI (0.025 g, 0.14 mmol), Pd(PPh_3_)_2_Cl_2_ (0.097 g, 0.14 mmol), PPh_3_ (0.036 g, 0.14 mmol), and Et_3_N (2 mL) in anhydrous DMF (3 mL) was stirred for 16 hours at 70°C under N_2_ atmosphere. The reaction mixture was partitioned between water (10 mL) and EtOAc (10 mL). The aqueous layer was washed with EtOAc (2×10 mL). The combined organic layer was dried over MgSO_4_, filtered and concentrated *in vacuo*. MPLC purification (Hex∶EtOAc/70∶30) of the residue gave **36y** as a pale yellow solid (0.20 g, 79%). ^1^H NMR (CDCl_3_) δ 9.74 (s, 1H), 7.65–7.63 (m, 4H), 7.35–7.32 (m, 4H), 7.28–7.27 (m, 2H), 7.23 (s, 1H), 4.75 (brs, 1H), 3.87 (s, 3H), and 3.78 (s, 3H); ^13^C NMR (CDCl_3_) δ 185.27, 160.96, 144.45, 128.71, 128.28, 127.25, 126.24, 125.29, 116.40, 103.82, 75.19, 74.83, 52.10, and 34.88.

*N*,*N*-Bis(7-(1,3-dioxoisoindolin-2-yl)heptyl)-4-formyl-5-(3-droxy-3,3-diphenylprop-1-ynyl))1-methyl–1*H*-pyrrole-2-carboxamide (**38y**). To a solution of **36y** (0.10 g, 0.3 mmol) in MeOH (10 mL) and water (4 mL) was added KOH (0.06 g, 1.1 mmol) and stirred at 60°C for 2 hours. The solvent was removed *in vacuo*. The residue obtained was acidified with 2 N HCl to pH 2–3 and extracted with EtOAc (10 mL). The aqueous layer was washed with EtOAc (2×10 mL). The combined organic layer was dried over MgSO_4_, filtered and concentrated *in vacuo* to give **37y** as a light brown solid. The crude product was subjected to the next reaction without further purification. A solution of **37y** (0.20 g, 0.6 mmol), **14x** (0.34 g, 0.7 mmol), BOP (0.49 g, 1.1 mmol), HOBt (0.15 g, 1.1 mmol) in anhydrous DMF (3 mL) and NMM (2 mL) was stirred at room temperature for 16 hours. The reaction mixture was partitioned between water and dichloromethane (20 mL). The aqueous layer was washed with dichloromethane (2×20 mL). The combined organic layer was dried over MgSO_4_, filtered and then concentrated *in vacuo*. MPLC purification (Hex∶EtOAc/50∶50) of the residue gave **38y** as a pale yellow oil. (0.30 g, 53%). ^1^H NMR (CDCl_3_) δ 9.82 (s, 1H), 7.78–7.66 (m, 4H), 7.62–7.58 (m, 8H), 7.27–7.24 (m, 4H), 7.20–7.15 (m, 2H), 6.55 (s, 1H), 5.36 (s, 1H), 3.62–3.50 (m, 7H), 3.34 (t, *J* = 6.8 Hz, 4H), 1.66–1.38 (m, 8H), and 1.36–1.04 (m, 12H); ^13^C NMR (CDCl_3_) δ 185.15, 168.66, 162.58, 144.92, 134.12, 132.21, 129.97, 128.53, 127.98, 127.28, 126.19, 125.49, 123.35, 108.30, 102.95, 74.90, 74.83, 49.20, 45.20, 38.04, 33.85, 31.60, 28.64, and 26.88.

(*E*)-Ethyl 3-(5-(bis(7-(1,3-dioxoisoindolin-2-yl)heptyl)carbamoyl)-2-(3-hydroxy-3,3-diphenylprop-1-ynyl)-1-methyl-1*H*-pyrrol-3-yl)acrylate (**39y**). To a stirred solution of **38y** (0.30 g, 0.4 mmol) in benzene (10 mL) was added (ethoxycarbonylmethylene)triphenylphosphorane (0.31 g, 0.9 mmol). The resulting mixture was stirred under reflux for 16 hours. The reaction mixture was cooled to room temperature and the solvent was removed *in vacuo*. MPLC purification (Hex∶EtOAc/50∶50) of the residue gave **39y** as a pale yellow oil (0.22 g, 68%). ^1^H NMR (CDCl_3_) δ 7.75–7.66 (m, 4H), 7.63–7.57 (m, 9H), 7.28–7.25 (m, 4H), 7.19–7.18 (m, 2H), 6.31 (s, 1H), 6.17 (d, *J* = 16.0 Hz, 1H), 4.82 (s, 1H), 4.03 (q, *J* = 6.8 Hz, 2H), 3.62–3.53 (m, 7H), 3.35 (t, *J* = 7.2 Hz, 4H), 1.64–1.48 (m, 8H), and 1.36–1.15 (m, 15H); ^13^C NMR (CDCl_3_) δ 168.64, 167.64, 163.06, 145.15, 136.84, 134.08, 132.20, 129.58, 128.49, 127.85, 126.25, 123.60, 123.32, 119.97, 115.60, 108.00, 102.65, 76.56, 75.07, 60.26, 49.20, 45.20, 38.03, 33.72, 28.86, 28.63, 26.90, and 14.58.

Ethyl 3-(5-(bis(7-(1,3-dioxoisoindolin-2-yl)heptyl)carbamoyl)-2-(3-hydroxy-3,3-diphenylpropyl)- 1-methyl-1*H*-pyrrol-3-yl)propanoate (**40y**). To a stirred solution of **39y** (0.11 g, 0.12 mmol) in EtOAc (10 mL) was added dry powdered 10% Pd-C (0.01 g). The resulting mixture was stirred at room temperature for 48 hours under a balloon of hydrogen. The reaction mixture was filtered through Celite and the filtrate was concentrated to give **40y** as a colorless oil (0.10 g, 90%). The crude product was subjected to the next reaction without further purification. ^1^H NMR (CDCl_3_) δ 7.82–7.80 (m, 4H), 7.70–7.68 (m, 4H), 7.46–7.44 (m, 4H), 7.32–7.20 (m, 6H), 6.03 (s, 1H), 4.04 (q, *J* = 6.8 Hz, 2H), 3.63 (t, *J* = 7.2 Hz, 4H), 3.44–3.36 (m, 7H), 2.60–2.52 (m, 4H), 2.46–2.39 (m, 4H), 2.04 (s, 1H), 1.68–1.60 (m, 4H), 1.58–1.49 (m, 4H), and 1.30–1.17 (m, 15H).

*N*,*N*-Bis(7-aminoheptyl)-5-(3-hydroxy-3,3-diphenylpropyl)-4-(3-(hydroxyamino)-3-oxopropyl)-1-methyl-1*H*-pyrrole-2-carboxamide (**11**). To a stirred solution of **40y** (0.10 g, 0.11 mmol) in MeOH∶THF (4 mL each) was added 50% aqueous hydroxylamine (4 mL) followed by KCN (0.04 g, 0.55 mmol ). The resulting mixture was stirred at 35°C for 24 hours. After the solvent was removed *in vacuo*, the residue was subjected to HPLC purification (eluting time: 40 minutes). The eluent from HPLC was concentrated *in vacuo* at room temperature to remove the organic solvents. The resulting aqueous solution was lyophilized to give **11•2TFA** as a white sticky solid (0.032 g, 33%). The semi-preparative and analytical HPLC retention times of **11•2TFA** are 16.52 and 11.35 minutes, respectively (see Figure S10 for chromatograms of **11•2TFA** before and after the HPLC purification). ^1^H NMR (DMSO-*d*_6_) δ 10.38 (brs, 1H), 7.71 (brs, 6H), 7.51–7.49 (m, 4H), 7.29–7.25 (m, 4H), 7.17–7.14 (m, 2H), 5.96 (s, 1H), 3.35–3.29 (m, 7H), 2.73–2.70 (m, 4H), 2.43 (m, 2H), 2.35 (m, 4H), 2.08 (t, *J* = 7.2 Hz, 2H), 1.48–1.42 (m, 8H), and 1.23–1.18 (m, 12H); ^13^C NMR (DMSO-*d*_6_) δ 169.47, 164.32, 158.98 (q), 148.65, 133.40, 128.54, 126.88, 126.36, 125.04, 117.84, 116.64 (q), 110.35, 76.85, 41.75, 39.47, 34.54, 32.05, 28.86, 27.58, 26.68, 26.39, 21.72, and 19.04; IR cm^−1^ 3428, 3085, 3056, 3023, 2934, 2856, 1679, 1597, 1495, 1201, and 1136; HRMS-ESI calculated for C_38_H_57_N_5_O_4_ [M+H^+^] 648.4483, found 648.4511.

#### Synthesis of AHP

1-Benzyl-*N*,*N*-bis(7-(1,3-dioxoisoindolin-2-yl)heptyl)-4-formyl-5-(3-hydroxy-3,3-diphenylprop-1-ynyl))-1*H*-pyrrole-2-carboxamide (**38z**). A solution of **37x** (0.49 g, 1.13 mmol), **14y** (0.68 g, 1.35 mmol), BOP (0.99 g, 2.25 mmol), HOBt (0.30 g, 2.25 mmol) in anhydrous DMF (3 mL) and NMM (2 mL) was stirred at room temperature for 16 hours. The reaction mixture was partitioned between water and CH_2_Cl_2_. The organic layer was dried over MgSO_4_, filtered, and concentrated *in vacuo*. MPLC purification (Hex∶EtOAc/50∶50) of the residue gave **38z** as a pale yellow oil. (0.72 g, 69%). ^1^H NMR (CDCl_3_) δ 9.90 (s, 1H), 7.82–7.74 (m, 4H), 7.68–7.64 (m, 4H), 7.56–7.54 (m, 4H), 7.28–7.17 (m, 9H), 7.03–7.00 (m, 2H), 6.59 (s, 1H), 5.36 (s, 2H), 4.37 (brs, 1H), 3.68–3.54 (m, 4H), 3.28–3.18 (m, 2H), 3.06–2.94 (m, 2H), 1.70–1.23 (m, 12H), and 1.18–0.91 (m, 8H); ^13^C NMR (CDCl_3_) δ 185.26, 168.69, 162.59, 144.52, 136.86, 134.11, 132.30, 129.55, 128.88, 128.61, 128.13, 127.84, 127.40, 126.19, 125.04, 123.39, 109.02, 102.66, 75.40, 75.13, 49.62, 38.11, 29.13, 28.69, 27.28, 27.09, 26.93, and 26.54.

(*E*)-Ethyl 3-(1-benzyl-5-bis(7-(1,3-dioxoisoindolin-2-yl)heptyl)carbamoyl)-2-(3-hydroxy-3,3-diphenylprop-1-ynyl)-1*H*-pyrrol-3-yl)acrylate (**39z**). To a stirred solution of **38z** (0.65 g, 0.71 mmol) in benzene (20 mL) was added (ethoxycarbonylmethylene)triphenylphosphorane (0.62 g, 1.78 mmol). The resulting mixture was stirred under reflux for 16 hours. The reaction mixture was cooled to room temperature and the solvent was removed *in vacuo*. MPLC purification (Hex∶EtOAc/50∶50) of the residue gave **39z** as a pale yellow oil (0.54 g, 77%). ^1^H NMR (CDCl_3_) δ 7.82–7.78 (m, 4H), 7.71–7.67 (m, 5H), 7.57–7.55 (m, 4H), 7.30–7.18 (m, 9H), 7.03–7.02 (m, 2H), 6.39 (s, 1H), 6.24 (d, *J* = 16.0 Hz, 1H), 5.32 (s, 2H), 4.17 (q, *J* = 7.2 Hz, 2H), 3.68–3.61 (m, 5H), 3.32–3.22 (m, 2H), 3.12–3.01 (m, 2H), 1.70–1.54 (m, 4H), and 1.46–1.00 (m, 19H); ^13^C NMR (CDCl_3_) δ 168.64, 167.62, 163.06, 145.11, 137.56, 136.88, 134.09, 132.21, 129.06, 128.70, 128.46, 127.79, 127.74, 126.28, 123.66, 123.32, 119.95, 115.76, 108.67, 102.81, 76.66, 75.02, 60.31, 49.38, 45.40, 38.06, 29.11, 28.64, 26.82, and 14.60.

Ethyl 3-(1-benzyl-5-(bis(7-(1,3-dioxoisoindolin-2-yl)heptyl)carbamoyl)-2-(3-hydroxy-3,3-diphenylpropyl)-1*H*-pyrrol-3-yl)propanoate (**40z**). To a stirred solution of **39z** (0.12 g, 0.12 mmol) in EtOAc (20 mL) was added 10% dry Pd-C (0.012 g). The resulting mixture was stirred at room temperature for 48 hours under a balloon of hydrogen. The reaction mixture was filtered through Celite and the filtrate was concentrated to give **40z** as a colorless oil. The crude product was subjected to the next reaction without further purification. ^1^H NMR (CDCl_3_) δ 7.85–7.79 (m, 4H), 7.72–7.67 (m, 4H), 7.36–7.31 (m, 4H), 7.28–7.22 (m, 4H), 7.20–7.11 (m, 5H), 6.78 (d, *J* = 7.2 Hz, 2H), 6.10 (s, 1H), 5.14 (s, 2H), 4.05 (q, *J* = 6.8 Hz, 2H), 3.64 (t, *J* = 7.2 Hz, 4H), 3.25 (t, *J* = 7.2 Hz, 4H), 2.63 (t, *J* = 7.6 Hz, 2H), 2.50–2.40 (m, 4H), 2.35–2.26 (m, 2H), 1.68–1.58 (m, 4H), and 1.42–1.08 (m, 19H).

*N*,*N*-Bis(7-aminoheptyl)-1-benzyl-5-(3-(hydroxy-3,3-diphenylpropyl)-4-(3-hydroxyamino)-3-oxopropyl))-1*H*-pyrrole-2-carboxamide (**AHP**). To a stirred solution of **40z** (0.14 g, 0.14 mmol) in MeOH∶THF (2 mL each) was added 50% aqueous hydroxylamine (4 mL) followed by KCN (0.04 g, 0.56 mmol). The resulting mixture was stirred at 35°C for 24 hours. After the solvent was removed *in vacuo*, the residue was subjected to HPLC purification (eluting time: 45 minutes). The eluent from HPLC was concentrated *in vacuo* at room temperature to remove the organic solvents. The resulting aqueous solution was lyophilized to give **AHP•2TFA** as a white sticky solid (0.034 g, 25%). The semi-preparative and analytical HPLC retention times of **AHP•2TFA** are 18.60 and 12.93 minutes, respectively (see Figure S11 for chromatograms of **AHP•2TFA** before and after the HPLC purification). ^1^H NMR (DMSO-*d*_6_) δ 10.41 (brs, 1H), 7.71 (brs, 6H), 7.42–7.38 (m, 4H), 7.26–7.10 (m, 9H), 6.67 (d, *J* = 7.2 Hz, 2H), 6.09 (s, 1H), 5.14 (s, 2H), 3.26–3.18 (m, 4H), 2.78–2.62 (m, 4H), 2.48–2.41 (overlapped with DMSO-*d_6_*, 2H), 2.35–2.28 (m, 4H), 2.11 (t, *J* = 7.2 Hz, 2H), 1.50–1.42 (m, 4H), 1.36–1.28 (m, 4H), 1.24–1.12 (m, 8H), and 1.11–1.04 (m, 4H); ^13^C NMR (DMSO-*d_6_*) δ 169.46, 164.21, 158.68 (q), 148.59, 140.08, 133.69, 128.91, 128.54, 127.39, 126.86, 126.44, 126.28, 124.93, 118.32, 117.57 (q), 111.44, 76.72, 55.61, 47.22, 42.32, 39.40, 34.57, 28.92, 27.58, 26.67, 26.34, 21.78, and 19.05; IR cm^−1^ 3416, 3085, 3056, 3023, 2934, 2860, 1687, 1614, 1495,1450, 1205, and 1136; HRMS-ESI calculated for C_44_H_61_N_5_O_4_ [M+H^+^] 724.4796, found 724.4791.

### In Vitro Evaluation of AHP

Assays of endopeptidase activities of BoNTAe and BoNTBe were done at 37°C and contained 0.5 mM substrate, 0.5–1.5 µg/mL recombinant BoNTAe or BoNTBe, 40 mM HEPES, and 0.05% tween at pH 7.3. BoNTAe inhibition assays also contained 1 mM dithiothreitol, 25 µM ZnCl_2_, and 0.5 mg/mL BSA, while BoNTBe inhibition assays were supplemented with 1 mM dithiothreitol only. Substrate for BoNTAe was an SNAP-25 fragment containing residues 187–203 with *N*- and *C*-termini acylated and amidated, respectively [51], while that for BoNTBe was residues 60–94 of VAMP [52]. Inhibitors were dissolved in dimethyl sulfoxide at 10 times the final assay concentration, then diluted into the assay mixture containing substrate, followed by addition of the endopeptidase (*i.e.*, inhibitor and endopeptidase were not preincubated). Assay times and endopeptidase concentrations were adjusted so that less than 10% of the substrate was hydrolyzed. Assays were stopped by acidification with TFA and analyzed by reverse-phase HPLC as described [34].

Inhibition of BoNTAe by **AHP** was determined in three independent experiments using nine concentrations of **AHP** in each. *K*_i_ was calculated from slopes of Dixon plots with the equation *K*_i_ = *K*_m_/[(slope)(V_max_)(S)], where (S) was the substrate concentration [35]. Kinetic constants for the substrate were taken from reference [36].

### MMDSs of the AHP•BoNTAe Complex

The atomic charges of **AHP** were obtained according to the RESP procedure [53] with *ab initio* calculations at the HF/6-31G*//HF/6-31G* level using the Gaussian 98 program [54]. The starting structure of **AHP**•BoNTAe was generated by (1) manually docking **AHP** into the BoNTAe active site as described in Section 2.2 and (2) replacing the zinc ion with the tetrahedral zinc ion of the cationic dummy atom approach [19], [43]–[45]. For BoNTAe, His223 and His227 were treated as HIN (histidinate) [44], [55], [56]; His39, His230, and His269 were treated as HID; all other His residues were treated as HIP; Glu261 and Glu351 were treated as GLH [44], [55], [56]. A total of 39 crystallographically determined water molecules (named HOH) located inside the enzyme were included for simulations. The topology and coordinate files of the water-containing **AHP**•BoNTAe complex were generated by the PREP, LINK, EDIT, and PARM modules of the AMBER 5.0 program [57]. The complex was refined by energy minimization using a dielectric constant of 1.0 and 100 cycles of steepest-descent minimization followed by 100 cycles of conjugate-gradient minimization. The refined complex was solvated with 12,098 TIP3P water molecules (named WAT) [58], leading to a system of 43,279 atoms. The WAT molecules were obtained from solvating the complex using a pre-equilibrated box of 216,000 TIP3P molecules, whose hydrogen atom charge was set to 0.4170, where any water molecule was removed if it had an oxygen atom closer than 2.2 Å to any solute atom or a hydrogen atom closer than 2.0 Å to any solute atom, or if it was located further than 9.0 Å along the x-, y-, or z-axis from any solute atom. The solvated complex system were energy-minimized for 100 cycles of steepest-descent minimization followed by 100 cycles of conjugate-gradient minimization to remove close van der Waals contacts in the system, then heated from 0 to 300 K at a rate of 10 K/ps under constant temperature and volume, and finally simulated independently with a unique seed number for initial velocities at 300 K under constant temperature and pressure using the PMEMD module of the AMBER 8.0 program [22] with the AMBER force field (ff99SB) [59], [60].

All simulations used (1) a dielectric constant of 1.0, (2) the Berendsen coupling algorithm [61], (3) a periodic boundary condition at a constant temperature of 300 K and a constant pressure of 1 atm with isotropic molecule-based scaling, (4) the Particle Mesh Ewald method to calculate long-range electrostatic interactions [62], (5) a time step of 1.0 fs, (6) the SHAKE-bond-length constraints applied to all the bonds involving the H atom, (7) saving the image closest to the middle of the “primary box” to the restart and trajectory files, (8) formatted restart file, and (9) default values of all other inputs of the PMEMD module. These simulations were carried out on a cluster of 300 Apple Xserves each equipped with two G5 processors at a clock rate of 2.0/2.3 GHz.

Average structures were obtained by using the CARNAL module of the AMBER 5.0 program. Cluster analyses were performed by using the PTRAJ module of the AMBER 10 program [22]. RMSDs were calculated by using the McLachlan algorithm [63] as implemented in the ProFit program (V2.6, http://www.bioinf.org.uk/software/profit/). The C&F RMSDs were obtained by (1) generating symmetry mates within 12 Å using PyMol 0.99rc6 [64], (2) identifying not-free residues that have a distance of <8.0 Å between any non-hydrogen atom of the structure in the primary cell and any non-hydrogen atom of the symmetry mates, (3) identifying hot residues that have alpha-carbon atoms with B factors greater than the average B factor for alpha carbons, (4) identifying short peptides with up to four residues that are between two hot residues, between two not-free residues, or between a hot and a not-free residue, (5) deleting the hot and not-free residues and the short peptides from the crystal structure and from the structure to be compared, and (6) computing the alpha-carbon RMSD of the truncated proteins using the ProFit program.

## Supporting Information

Figure S1 Synthesis toward Analog 2.
(0.21 MB PDF)

Figure S2 Synthesis toward Analog 6.
(0.07 MB PDF)

Figure S3 Synthesis of Analog 3.
(0.10 MB PDF)

Figure S4 Synthesis of Analog 4.
(0.08 MB PDF)

Figure S5 Synthesis of Analog 5.
(0.09 MB PDF)

Figure S6 Synthesis of Analog 7.
(0.07 MB PDF)

Figure S7 Chromatograms of 8•2TFA before and after the HPLC purification.
(6.12 MB PDF)

Figure S8 Chromatograms of 9•2TFA before and after the HPLC purification.
(6.17 MB PDF)

Figure S9 Chromatograms of 10b•2TFA before and after the HPLC purification.
(6.11 MB PDF)

Figure S10 Chromatograms of 11•2TFA before and after the HPLC purification.
(6.45 MB PDF)

Figure S11 Chromatograms of AHP•2TFA before and after the HPLC purification.
(6.03 MB PDF)

Dataset S1 The average conformation of AHP-bound BoNTAe in Cluster 3 of the second-round MMDSs.
(0.38 MB TXT)

Dataset S2 The representative conformation of AHP-bound BoNTAe in Cluster 3 of the second-round MMDSs.
(0.60 MB TXT)
