# Supplementary material for: Potent New Small-Molecule Inhibitor of Botulinum Neurotoxin Serotype A Endopeptidase Developed by Synthesis-Based Computer-Aided Molecular Design
Source: PLoS One. 2009 Nov 10;4(11):e7730. doi: 10.1371/journal.pone.0007730 (PMC2771286; doi:10.1371/journal.pone.0007730)
Supplement: Figure S1 — Synthesis toward Analog 2. (0.21 MB PDF) [file pone.0007730.s001.pdf]

Figure S1

Synthesis toward Analog 2

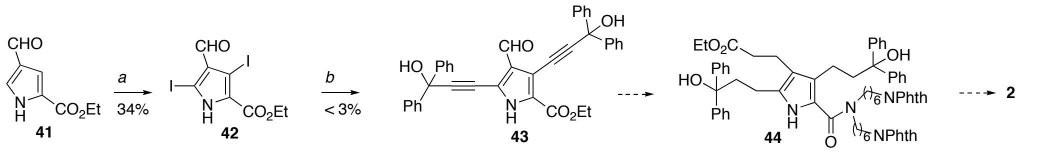

a. $CF_3COOAg/I_2/CHCl_3$/0 °C to rt, 18 h; b. 1,1-diphenylprop-2-yn-1-ol, $Pd(PPh_3)_2Cl_2$, PPh_3, CuI, Et_3N, DMF, 100 °C,24h

Ethyl 4-formyl-3,5-diiodo-1*H*-pyrrole-2-carboxylate (42)[1]. To a stirred solution of **41** [2] (0.70 g, 4.16 mmol) in $CHCl_3$ (20 mL) at 0 °C was added CF_3CO_2Ag (2.75 g, 12.47 mmol) followed by I_2 (1.58 g, 12.47 mmol). The resulting mixture was stirred for 18 hours in dark at room temperature. The reaction mixture was filtered through Celite and the filtrate was washed with saturated aqueous solution of $Na_2S_2O_3$. The organic layer was dried over anhydrous $MgSO_4$, filtered and concentrated *in vacuo*. MPLC purification (CH_2Cl_2:Et_2O/95:5) of the residue gave **42** as a white solid (0.60 g, 34%). ^1H NMR (DMSO-d_6) δ 13.58 (brs, 1H), 9.56 (s, 1H), 4.28 (q, *J*=7.2 Hz, 2H), and 1.32 (t, *J*=7.2 Hz, 3H).

Ethyl 4-formyl-3,5-bis(3-hydroxy-3,3-diphenylprop-1-ynyl)-1*H*-pyrrole-2-carboxylate (43). A solution of **42** (0.05 g, 0.12 mmol), 1,1-diphenylprop-2-yn-1-ol (0.075 g, 0.36 mmol), CuI (0.005 g, 0.026 mmol), $Pd(PPh_3)_2Cl_2$ (0.009 g, 0.0128 mmol), PPh_3 (0.007 g, 0.027 mmol), and Et_3N (1 mL) in anhydrous DMF (1 mL) was stirred for 24 hours at 100 °C under N_2 atmosphere. The reaction mixture was partitioned between water and EtOAc. The combined organic layer was dried over $MgSO_4$, filtered and concentrated *in vacuo*. MPLC purification (Hex:EtOAc/20:80) of the residue gave **43** in less than 3% yield with inseparable impurities (see the attached ^1H NMR spectrum).^1H NMR ($CDCl_3$) δ 10.25 (brs, 1H), 9.92 (s, 1H), 7.70-7.60 (m, 2H), 7.64-7.60 (m, 2H), 7.36-7.22 (m, 16H), 4.28 (q, *J*=7.2 Hz, 2H), and 1.32 (t, *J*=7.2 Hz, 3H).

References

1. Farnier M, Fournari P (1973) Synthese d'iodoaldehydes Pyrroliques. Bulletin de la Societe Chimique de France: 351-356.

2. Elliott LD, Berry M, Orr-Ewing AJ, Booker-Milburn KI (2007) The Intramolecular Photometathesis of Pyrroles. Journal of the American Chemical Society 129: 3078-3079.

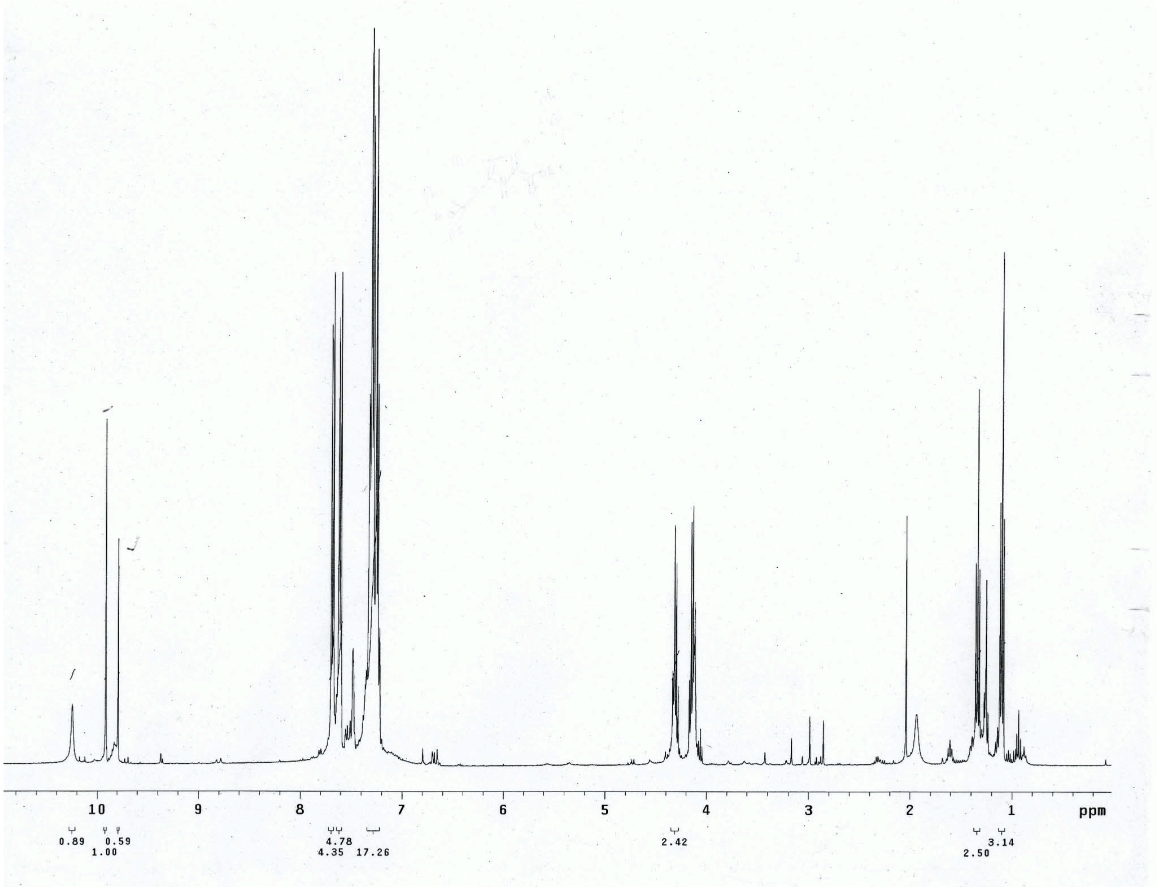

^{1}H NMR Spectrum of Compound 43 with inseparable impurities
