# Supplementary material for: Potent New Small-Molecule Inhibitor of Botulinum Neurotoxin Serotype A Endopeptidase Developed by Synthesis-Based Computer-Aided Molecular Design
Source: PLoS One. 2009 Nov 10;4(11):e7730. doi: 10.1371/journal.pone.0007730 (PMC2771286; doi:10.1371/journal.pone.0007730)
Supplement: Figure S2 — Synthesis toward Analog 6. (0.07 MB PDF) [file pone.0007730.s002.pdf]

Figure S2

Synthesis toward Analog 6

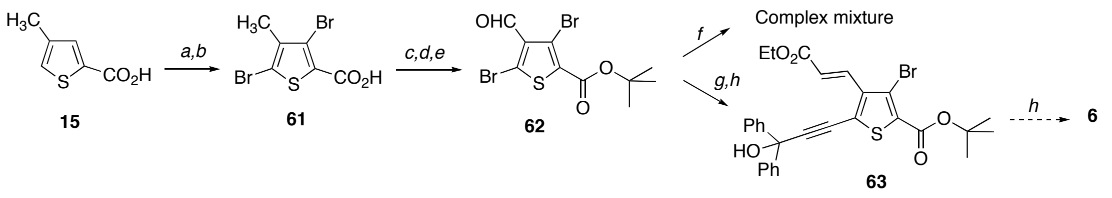

a. HCl/MeOH, reflux; *b.* Br_2 (neat), rt, 18 h; *c.* t-BuOH/EDCI/DMAP/CH_2Cl_2, rt, 18 h; *d.* NBS/AIBN/CCl_4, reflux, 5 h; *e.* 2-Nitropropane/NaOEt/EtOH, rt, 16 h; *f.* 1,1-Diphenylprop-2-yn-1-ol (excess)/CuI/$PdCl_2(PPh_3)_2$/K_2CO_3/DMF; *g.* $EtO_2CCH{=}PPh_3$/benzene, reflux, 17 h; *h.* 1,1-Diphenylprop-2-yn-1-ol (excess)/CuI/$PdCl_2(PPh_3)_2$/Et_3N, rt.

3,5-Dibromo-4-methylthiophene-2-carboxylic acid (61). To a stirred solution of **15** (1.42 g, 10.0 mmol) in MeOH (20 mL) was added conc. HCl (2.0 mL), the resulting mixture was heated under reflux for 24 hours. The solvent was removed *in vacuo*, the residue neutralized with saturated aqueous $NaHCO_3$ solution, extrated with EtOAc, dried over $MgSO_4$, filtered, concentrated, and then purified by MPLC to give methyl ester of **15** as a colorless oil (1.25 g, 80%). ^1H NMR ($CDCl_3$) δ 7.60 (s, 1H), 7.14 (s, 1H), 3.87 (s, 3H), 2.28 (s, 3H). To this ester (0.38 g, 2.43 mmol) was added bromine (0.6 mL), the resulting mixture was stirred overnight (18 hours) at room temperature. The excess bromine was removed by blowing nitrogen into the reaction mixture. The resulting grey powder was triturated with Hex to give **61** as a white powder (0.67 g, 92%). ^1H NMR ($CDCl_3$) δ 2.28 (s, 3H); LREIMS 300, 299 ([M^+], 100%).

***tert*-Butyl 3,5-dibromo-4-formylthiophene-2-carboxylate (62).** To a stirred solution of **61** (0.60 g, 2.00 mmol), DMAP (0.24 g, 2.00 mmol), EDCI (0.46 g, 2.40 mmol) in anhydrous CH_2Cl_2 (7 mL) was added *tert*-BuOH (0.23 mL, 2.40 mmol) at room temperature. After overnight stirring the solvent was removed *in vacuo*, the residue was purified by MPLC (gradient Hex to EtOAc) to give *tert*-butyl 3,5-dibromo-4-methylthiophene-2-carboxylate as a colorless oil (0.61 g, 86%). ^1H NMR ($CDCl_3$) δ 2.24 (s, 3H) and 1.57 (s, 9H); ^{13}C NMR ($CDCl_3$) δ 159.43, 139.49, 117.93, 115.90, 83.25, 28.43, and 16.33. This *tert*-butyl ester (0.61 g, 1.71 mmol) and a few crystals of

AIBN in CCl_4 (10 mL) was added NBS (0.34 g, 1.99 mmol) at room temperature. The mixture was heated under reflux for 5 hours. The solvent was removed *in vacuo*, the residue was purified by MPLC (gradient Hex to EtOAc) to give 0.74 (100%) of *tert*-butyl 3,5-dibromo-4-(bromomethyl)thiophene-2-carboxylate as a light yellow oil (0.74 g, 100%). ^{1}H NMR ($CDCl_3$) δ 4.49 (s, 2H) and 1.58 (s, 9H). To a freshly prepared alcoholic NaOEt (from Na metal 39 mg, 1.70 mmol in 2 mL EtOH) solution 2-nitropropan (199 μL, 2.21 mmol) was added at room temperature. To this was added a solution of *tert*-butyl 3,5-dibromo-4-(bromomethyl)thiophene-2-carboxylate (0.74 g, 1.70 mmol) in EtOH (2 mL) at room temperature. The resulting mixture was stirred at room temperature for 16 hours. The solvent was removed *in vacuo* and the residue was dissolved in EtOAc (10 mL), washed with water (5 mL), dried over $MgSO_4$, filtered, concentrated, and then purified by MPLC to give **62** as a white solid (0.30 g, 47%). ^{1}H NMR ($CDCl_3$) δ 10.02 (s, 1H) and 1.59 (s, 9H); ^{13}C NMR ($CDCl_3$) δ 185.19, 158.58, 134.30, 131.28, 128.16, 116.79, 84.49, and 28.40.

(*E*)-*tert*-Butyl 3-bromo-4-(3-ethoxy-3-oxoprop-1-enyl)-5-(3-hydroxy-3,3-diphenylprop-1-ynyl)thiophene-2-carboxylate (63). The compound **62** (50 mg, 0.14 mmol) and (ethoxycarbomethylene)triphenylphosphorane (0.15 g, 0.42 mmol) in benzene (2 mL) was heated at 80 °C for 3 hours. The solvent was removed *in vacuo*, the residue purified by MPLC (gradient Hex to EtOAc) to give (*E*)-*tert*-butyl 3,5-dibromo-4-(3-ethoxy-3-oxoprop-1-enyl)thiophene-2-carboxylate as a white solid (0.06 g, 98%). ^{1}H NMR ($CDCl_3$) δ 7.60 (d, *J* = 16.4 Hz, 1H), 6.85 (d, *J* = 16.4 Hz, 1H), 4.28 (q, *J* = 7.1 Hz, 2H), 1.57 (s, 9H), and 1.34 (t, *J* = 7.1 Hz, 3H). A 7 mL vial was charged with the above compound (44 mg, 0.10 mmol), 1,1-diphenylprop-2-yn-1-ol (21 mg, 0.50 mmol), CuI (2.7 mg, 0.014 mmol), $PdCl_2(PPh_3)_2$ (4.9 mg, 7.0×10^{-6} mol) and Et_3N (2 mL) under N_2. While stirring the mixture was degassed 15 minutes by passing N2 through the solution. The vial was tightly capped, heated at 60 °C for 3 hours. After insoluble materials were filtered off (Celite), concentrated *in vacuo*, the residue was purified by MPLC (gradient Hex to EtOAc) to give **63** as a yellow solid foam (0.045 g, 79%).

^{1}H NMR ($CDCl_3$) δ 7.75 (d, *J* = 16.2 Hz, 1H), 7.60 (d, *J* = 7.6 Hz, 4H), 7.38–7.30 (m, 6H), 6.99 (d. *J* = 16.2 Hz, 1H), 4.22 (q, *J* = 7.1 Hz, 2H), 3.26 (s, 1H), 1.58 (s, 9H), and 1.29 (t, *J* = 7.1 Hz, 3H); ^{13}C NMR ($CDCl_3$) δ 166.89, 159.08, 143.99, 139.17, 135.54, 129.96, 128.78, 128.64, 128.36, 128.32, 126.28, 125.45, 123.34, 117.93, 104.42, 83.93, 79.25, 75.47, 61.01, 28.41, and 14.54.
