# Supplementary material for: Potent New Small-Molecule Inhibitor of Botulinum Neurotoxin Serotype A Endopeptidase Developed by Synthesis-Based Computer-Aided Molecular Design
Source: PLoS One. 2009 Nov 10;4(11):e7730. doi: 10.1371/journal.pone.0007730 (PMC2771286; doi:10.1371/journal.pone.0007730)
Supplement: Figure S3 — Synthesis of Analog 3. (0.10 MB PDF) [file pone.0007730.s003.pdf]

Figure S3

Synthesis of Analog 3

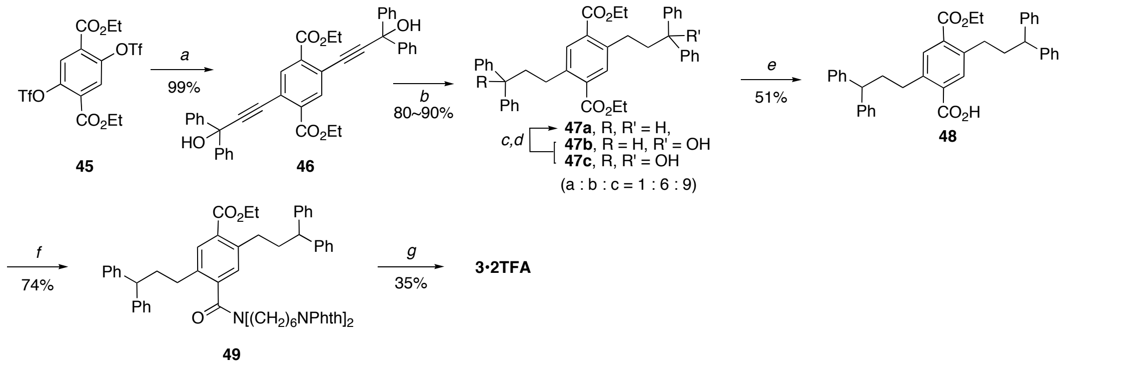

a. 1,1-Diphenylprop-2-yn-1-ol/CuI/$PdCl_2(PPh_3)_2$/Hunig's Base/DMF, rt, 18 h; *b.* H_2/Raney-Ni/MeOH, rt, 2 h; *c.* $InCl_3$/$ClPh_2SiH$/CH_2Cl_2, 45 min; *d.* H_2/Pd-C/EtOAc; *e.* KOH/EtOH, 80 °C, 16 h; *f.* $HN[(CH_2)_6NPhth]_2$/BOP/HOBt//NMM/DMF, rt, 16 h; *g.* 50 % aq. NH_2OH /KCN/MeOH/THF, 80 °C,36 h, HPLC purification using MeCN/ H_2O/TFA

Diethyl 2,5-bis(3-hydroxy-3,3-diphenylprop-1-ynyl)terephthalate (46). The titled intermediate was synthesized following the known method[1], briefly, a solution of CuI (80 mg, 0.42 mmol) and $PdCl_2(PPh_3)_2$ (0.15 g, 0.21 mmol) in anhydrous DMF (6 mL) and diisopropylethylamine (DIEA, 2.4 mL) was degassed by purging with nitrogen for 15 minutes. To this was added diethyl 2,5-bis(trifluoromethylsulfonyloxy)terephthalate (1.56 g, 3.00 mmol, prepared according to the literatre[2] and 1,1-diphenylprop-2-yn-1-ol (1.37 g, 6.60 mmol) at room temperature under nitrogen. After 17 hours stirring at room temperature, the reaction mixture was diluted with H_2O (10 mL), extracted with EtOAc (3 × 20 mL). The organic layer was stirred with decolorizing carbon (2 g) for 10 minutes, filtered through a Celite pad to give a red filtrate. The filtrated was concentrated and purified by recrystallization from (EtOAc-Hex) to afford 0.70 g of white needles. The mother liquor was concentrated and purified by MPLC (gradient Hex to EtOAc) to give additional 1.18 g of the product (total 1.88 g, 99%). ^{1}H NMR ($CDCl_3$) δ 8.11 (s, 2H), 7.71–7.68 (m, 8H), 7.37–7.34 (m, 8H), 7.30–7.27 (m, 4H), 4.31 (q, *J* = 7.2 Hz, 4H), 3.38 (br s, 2H), and 1.29 (t, *J* = 7.2 Hz, 6H); ^{13}C NMR ($CDCl_3$) δ 164.99, 144.86, 136.04, 134.94, 128.57, 128.04, 126.38, 122.74, 99.41, 84.86, 75.27, 62.22, and 14.42.

Diethyl 2,5-bis(3,3-diphenylpropyl)terephthalate (47a). Raney-Ni (0.5 mL, slurry in water) was washed with MeOH (3 × 2 mL), covered with 5 mL of MeOH. To this was added compound **2** (140 mg, 0.22 mmol) .The resulting mixture was stirred at room temperature for 2 hours under a balloon of hydrogen. The aliquot was taken using a pipette, and the catalyst was washed several times with EtOAc. The washings and the aliquot were combined, concentrated *in vacuo*, and then purified by MPLC (gradient Hex to EtOAc) to give a mixture of **47a**, **47b** and **47c** at the ratio of 1 (6 mg): 6 (39 mg): 9 (63 mg) (in 80-90% yield). The ratio varies batch by batch, but 3a was always the least. **47a**: ^{1}H NMR (400 MHz, $CDCl_3$) δ 7.59 (s, 2H), 7.30–7.16 (m, 20H), 4.29 (q, *J* = 7.0 Hz, 4H), 3.96 (t, *J* = 7.6 Hz, 2H), 2.89–2.85 (m, 4H), 2.38–2.32 (m, 4H), and 1.33 (t, *J* = 7.0 Hz, 6H); ^{13}C NMR (100 MHz, $CDCl_3$) δ 167.48, 144.96, 141.08, 133.11, 133.01, 128.69, 128.14, 126.41, 61.40, 51.55, 37.62, 32.67, and 14.51. **Diethyl 2-(3,3-diphenylpropyl)-5-(3-hydroxy-3,3-diphenylpropyl)terephthalate (47b)**: ^{1}H NMR ($CDCl_3$) δ 7.65 (s, 1H), 7.62 (s, 1H), 7.52–7.50 (m, 4H), 7.34–7.19 (m, 16H), 4.37–4.27 (m 4H), 3.97 (t, *J* = 7.8 Hz, 1H), 3.39 (br s, 1H), 2.92–2.87 (m, 4H), 2.64–2.58 (m 2H), 2.40–2.34 (m, 2H), 1.43–1.32 (m, 6H). **Diethyl 2,5-bis(3-hydroxy-3,3-diphenylpropyl)terephthalate (47c):** ^{1}H NMR ($CDCl_3$) δ 7.65 (s, 2H), 7.51–7.49 (m, 8H), 7.34–7.30 (m, 8H), 7.24–7.20 (m, 4H), 4.32 (q, *J* = 7.0 Hz, 4H), 3.36 (br s, 2H), 2.90–2.86 (m, 4H), 2.63–2.58 (m, 4H), and 1.36 (t, *J* = 7.0 Hz, 6H); ^{13}C NMR ($CDCl_3$) δ 167.28, 147.34, 142.04, 133.53, 132.66, 128.34, 126.94, 126.27, 61.72, 44.13, 28.91, and 14.53.

Both **47b** and **47c** were (individually or as a mixture of the two) treated with $InCl_3$ (5 mol%) in the presence of Chlorodiphenylsilane ($ClPh_2SiH$, 2 eq) in CH_2Cl_2 at room temperature for 30–45 minutes to dehydrate and give mono- and di-olefinic intermediate, respectively. The conditions were applied following the literature procedure.[3] The mono-olefinic intermediate (diethyl 2-(3,3-diphenylallyl)-5-(3,3-diphenylpropyl)terephthalate): ^{1}H NMR ($CDCl_3$) δ 7.67 (d, *J* = 1.4 Hz, 1H), 7.63 (d, *J* = 1.6 Hz, 1H), 7.43–7.17 (m, 20H), 6.26 (dt, *J* = 1.6, 7.2 Hz, 1H),

4.37–4.27 (m, 4H), 3.98 (t, *J* = 7.8 Hz, 1H), 3.79 (d, *J* = 7.2 Hz, 2H), 2.92–2.88 (m, 2H), 2.40–2.35 (m, 2H), and 1.39–1.32 (m, 6H); ^{13}C NMR ($CDCl_3$) δ 167.48, 167.34, 144.97, 142.91, 142.65, 141.38, 140.04, 139.71, 133.15, 133.08, 132.95, 132.89, 130.19, 128.71, 128.55, 128.33, 128.16, 127.68, 127.59, 127.42, 127.30, 126.43, 61.52, 61.38, 51.55, 37.62, 34.02, 32.68, 14.53, and 14.49. The di-olefinic intermediate (diethyl 2,5-bis(3,3-diphenylallyl)terephthalate): ^{1}H NMR ($CDCl_3$) δ 7.69 (s, 2H), 7.43–7.22 (m, 20H), 6.27 (t, *J* = 7.2 Hz, 2H), 4.33 (q, *J* = 7.0 Hz, 4H), 3.81 (d, *J* = 7.2 Hz, 4H), 1.36 (t, *J* = 7.0 Hz, 6H); ^{13}C NMR ($CDCl_3$) δ 167.36, 142.94, 142.63, 140.04, 139.98, 134.57, 133.06, 132.84, 130.20, 128.56, 128.33, 127.63, 127.59, 127.43, 127.31, 61.49, 33.99, and 14.49. These intermediates were stirred under hydrogen (balloon) in EtOAc to give 3a in 89% yield over two steps.

2,5-Bis(3,3-diphenylpropyl)-4-(ethoxycarbonyl)benzoic acid (48). The compounds **47a** (173 mg, 0.28 mmol) was dissolved in EtOH (5 mL), to this was added potassium hydroxide (32 mg, 2.0 eq), and then heated at 80 °C for 14 hours. After cooling to room temperature, the solvent was removed *in vacuo*, the residue diluted with H_2O (5 mL), acidified with 1 N HCl to pH 3, and then extracted with EtOAc. The organic layer was washed with brine, dried over $MgSO_4$, concentrated in vacuo, and then purified by MPLC (gradient Hex to EtOAc) to afford the title compound (84 mg, 51%). ^{1}H NMR ($CDCl_3$) δ 7.72 (s, 1H), 7.53 (s, 1H), 7.72–7.03 (m, 20H), 4.33–4.30 (m, 2H), 3.84–3.76 (m, 2H), 2.82–2.74 (m, 4H), 2.21–2.06 (m, 4H), and 1.35 (m, 3H).

Ethyl 4-(bis(6-(1,3-dioxoisoindolin-2-yl)hexyl)carbamoyl)-2,5-bis(3,3-diphenylpropyl)benzoate (49). To a stirred solution of compound **48** (18.9 mg, 3.24×10^{-5} mol), **14x** (15.4 mg, 3.24×10^{-5} mol), BOP (21.5 mg, 4.87×10^{-5} mol), and HOBt (6.6 mg, 4.87×10^{-5} mol) in 1 mL DMF was added NMM (5.3 μL, 4.87×10^{-5} mol) at room temperature. After stirring overnight at the same temperature, diluted with H_2O (2 mL), extracted with EtOAc (3 × 3 mL). The extracts were combined, washed with brine, dried over $MgSO_4$, concentrated, and then purified by

MPLC (gradient Hex to EtOAc) to afford **49** (0.025 g,74%). ^{1}H NMR ($CDCl_3$) δ 7.85–7.81 (m, 4H), 7.71–7.69 (m, 4H), 7.66 (s, 1H), 7.26–7.22 (m, 16H), 7.15–7.12 (m, 4H), 6.92 (s, 1H), 4.27 (q, J = 7.0 Hz, 2H), 3.96 (t, J = 7.7 Hz, 1H), 3.91 (t, J = 7.7 Hz, 1H), 3.71 (t, J = 7.2 Hz, 2H), 3.52 (t, J = 7.2 Hz, 2H), 3.48 (m, 1H), 3.22–3.17 (m, 1H), 2.96–2.72 (m, 4H), 2.47 (m, 1H), 2.35–2.25 (m, 3H), 1.73–1.69 (m, 2H), 1.52–1.30 (m, 15H), and 1.12–0.97 (m, 4H); ^{13}C NMR ($CDCl_3$) δ 170.17, 168.69, 168.57, 167.61, 145.05, 144.98, 144.48, 141.83, 140.29, 136.06, 134.13, 132.38, 132.32, 131.76, 130.31, 128.69, 128.67, 128.46, 128.17, 128.07, 128.02, 126.41, 126.37, 123.42, 123.40, 61.22, 51.78, 51.55, 48.51, 44.40, 38.13, 37.85, 36.63, 32.96, 31.43, 28.82, 28.55, 27.32, 26.96, 26.84, 26.57, 26.43, and 14.55.

N^1,N^1-Bis(6-aminohexyl)-2,5-bis(3,3-diphenylpropyl)-N^4-hydroxyterephthalamide (**3**). To a stirred solution of the compound 5 (25 mg, 2.40 × 10^{-5} mol) in 0.5 mL each of THF and MeOH was added 2 crystals of KCN at room temperature. The mixture was heated at 80 °C for 36 hours. After cooling to room temperature, the mixture was concentrated, purified by HPLC to give **3. 2TFA** (8.4 mg, 35%). Analytical retention time was 14.18 minutes for the product. ^{1}H NMR (DMSO-d_6) δ 13.00 (brs, 2H), 7.70–7.62 (m, 2H), 7.62 (s, 1H), 7.27–7.15 (m, 20H), 6.92 (s, 1H), 3.96–3.90 (m 2H), 3.54 (brs, 6H), 3.42–3.40 (m, 2H), 3.15 (m, 2H), 2.80–2.74 (m, 4H), 2.57–2.54 (m, 2H), 2.36–2.21 (m, 6H), 1.56–1.52 (m, 2H), 1.40–1.22 (m, 10H), and 0.97–0.92 (m, 4H); ^{13}C NMR (DMSO-d_6) δ 169.53, 169.13, 145.55, 141.29, 136.06, 131.19, 129.13, 128.23, 126.80, 51.49, 48.44, 44.29, 43.64, 39.25, 37.61, 36.47, 32.61, 31.38, 28.61, 27.78, 27.48, 27.32, 26.77, 26.30, 26.27, 26.06, and 23.33.

References

1. Sorensen JK, Vestergaard M, Kadziola A, Kilsa K, Nielsen MB (2006) Synthesis of Oligo(phenyleneethylene)-Tetrathiafulvalene Cruciforms for Molecular Electronics. Organic Letters 8: 1173-1176.

2. Zhang Q, Shi C, Zhang H-R, Wang KK (2000) Synthesis of 6H-Indolo[2,3-b][1,6]naphthyridines and Related Compounds as the 5-Aza Analogues of Ellipticine Alkaloids. The Journal of Organic Chemistry 65: 7977-7983.

3. Yasuda M, Onishi Y, Ueba M, Miyai T, Baba A (2001) Direct Reduction of Alcohols: Highly Chemoselective Reducing System for Secondary or Tertiary Alcohols Using Chlorodiphenylsilane with a Catalytic Amount of Indium Trichloride. The Journal of Organic Chemistry 66: 7741-7744.
