# Supplementary material for: Potent New Small-Molecule Inhibitor of Botulinum Neurotoxin Serotype A Endopeptidase Developed by Synthesis-Based Computer-Aided Molecular Design
Source: PLoS One. 2009 Nov 10;4(11):e7730. doi: 10.1371/journal.pone.0007730 (PMC2771286; doi:10.1371/journal.pone.0007730)
Supplement: Figure S4 — Synthesis of Analog 4. (0.08 MB PDF) [file pone.0007730.s004.pdf]

## Figure S4

### Synthesis of Analog 4

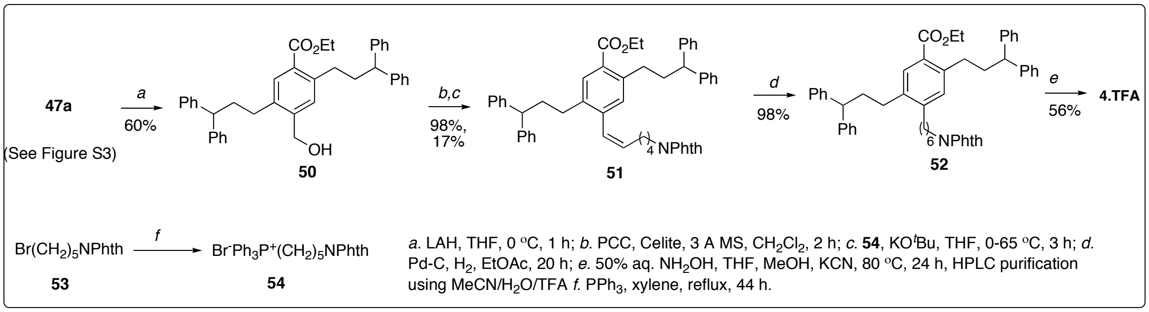


**Ethyl 2,5-bis(3,3-diphenylpropyl)-4-(hydroxymethyl)benzoate (50).** To a stirred solution of **47a (See Figure S3)** (0.028 g, 0.46 mmol in anhydrous THF (8 ml), at 0 °C was added $LiAlH_4$ (0.014 g, 0.37 mmol). The resulting mixture was stirred for 1 hour at the same temperature. The reaction mixture was quenched with sodium sulphate decahydrate, filtered and the filtrate was concentrated *in vacuo*. MPLC purification (Hex:EtOAc/80:20) of the residue gave **50** as a white solid (0.16 g, 60%). $^1H$ NMR ($CDCl_3$) δ 7.63 (s, 1H), 7.31–7.24 (m, 16H), 7.21–7.15 (m, 4H), 7.14 (s, 1H), 4.53 (d, *J* = 6.0 Hz, 2H), 4.26 (q, *J* = 7.2 Hz, 2H), 3.96 (q, *J* = 8.0 Hz, 2H), 2.89–2.84 (m, 2H), 2.60–2.54 (m, 2H), 2.36–2.29 (m, 4H), 1.31 (t, *J* = 8.0 Hz, 3H).

**Ethyl 4-(6-(1,3-dioxoisoindolin-2-yl)hexyl)-2,5-bis(3,3-diphenylpropyl)benzoate (52).** A solution of 50 (0.15 g, 0.26 mmol), PCC (pyridinium chromyl chloride) (0.068 g, 0.31 mmol), 3 A° molecular sieves (0.20 g) and Celite (0.20 g) in anhydrous $CH_2Cl_2$ (10 mL) was stirred at room temperature for 2 hours. The reaction mixture was filtered over a bed of silica gel. The solvent was removed from the filtrate to give the desired aldehyde in quantitative yield (0.15 g). The residue obtained was subjected to the next reaction without further purification.

To a solution of above aldehyde (0.15 g, 0.27 mmol) in anhydrous THF (10 mL), at 0 °C was added triphenyl (5-phthalimidopentyl)phosphonium bromide (**54**) (0.23 g, 0.41 mmol) and

KO$^t$Bu (0.04 g, 0.33 mmol). The resulting mixture was stirred at the same temperature for 30 minutes followed by 1 hour at room temperature and finally refluxed for 11 hours[1] The reaction mixture was partitioned between water (10 mL) and dichloromethane (10 mL). The organic layer was concentrated under vacuum to give a residue which was subjected to MPLC purification (Hex:EtOAc/80:20) to give **51** as an inseparable mixture of *E* and Z isomers (0.04 g, 17%).

To a stirred solution of **51** (0.04 g, 0.05 mmol) in EtOAc (10 mL) was added 10% dried Pd-C (0.003 g). The resulting mixture was stirred for 20 hours at room temperature under a balloon of hydrogen. The reaction mixture was filtered through Celite and the filtrate was concentrated to give **52** in quantitative yields (0.04 g). The crude material was subjected to next reaction without any further purification. $^1$H NMR ($CDCl_3$) δ 7.78–7.74 (m, 2H), 7.64–7.61 (m, 2H), 7.54 (s, 1H), 7.28–7.18 (m, 16H), 7.15–7.07 (m, 4H), 6.76 (s, 1H), 4.18 (q, *J* = 7.2 Hz, 2H), 3.89–3.87 (m, 2H), 3.59 (t, *J* = 7.2 Hz, 2H), 2.79–2.75 (m, 2H), 2.45–2.43 (m, 2H), 2.32–2.21 (m, 4H), 1.59–1.53 (m, 2H), 1.32 (m, 2H), and 1.28–1.15 (m, 9H).

**4-(6-Aminohexyl)-2,5-bis(3,3-diphenylpropyl)-N-hydroxybenzamide (4).** To a stirred solution of **52** (0.04 g, 0.04 mmol) in MeOH:THF (1:1, 4 mL) was added 50% aqueous hydroxylamine (1 mL) followed by the addition of catalytic amount of KCN. The resulting mixture was stirred at 80 °C for 24 hours. After the solvent was removed *in vacuo*, the residue was subjected to HPLC purification, which gave **4•TFA** as a colorless oil (0.02 g, 22%). Both semi-preparative and analytical HPLC retention time of **4•TFA** was 20.92 minutes. $^1$H NMR (400 MHz, $CD_3OD$) δ 7.67 (s, 1H), 7.32–7.28 (m, 8H), 7.27–7.24 (m, 8H), 7.18–7.12 (m, 4H), 6.84 (s, 1H), 3.96–3.92 (m, 2H), 2.88–2.82 (m, 4H), 2.57–2.53 (m, 2H), 2.41 (t, *J* = 8.0 Hz, 2H), 2.33–2.27 (m, 4H), 1.60–1.56 (m, 2H), 1.43–1.39 (m, 2H), and 1.31–1.25 (m, 4H).

**Triphenyl (5-phthalimidopentyl)phosphonium bromide (54).** To a solution of **53** (0.89 g, 3.00 mmol) in xylene (20 mL) was added triphenyl phosphine (0.79 g, 3.00 mmol). The reaction mixture was heated under reflux for 44 hours to give a white solid. The resultant mixture was filtered and washed with ether to give **54** as a white solid (0.15 g)[1]. $^1$H NMR (400 MHz, $CDCl_3$) δ 7.83–7.68 (m, 19H), 3.72 (m, 2H), 3.55 (m, 2H), 2.19 (m, 2H), and 1.67 (m, 4H).

## References


1. Olsen, D.K., et al., *Preparatioio and Stereochemistry of 4-Aryl-3-butenylamines. A Novel Synthesis of an Oxaz:olo[2,3-a Iisoindole.* The Journal of Organic Chemistry, 1980. **45**: p. 4049-4052.
