# Supplementary material for: Potent New Small-Molecule Inhibitor of Botulinum Neurotoxin Serotype A Endopeptidase Developed by Synthesis-Based Computer-Aided Molecular Design
Source: PLoS One. 2009 Nov 10;4(11):e7730. doi: 10.1371/journal.pone.0007730 (PMC2771286; doi:10.1371/journal.pone.0007730)
Supplement: Figure S5 — Synthesis of Analog 5. (0.09 MB PDF) [file pone.0007730.s005.pdf]

Figure S5

Synthesis of Analog 5

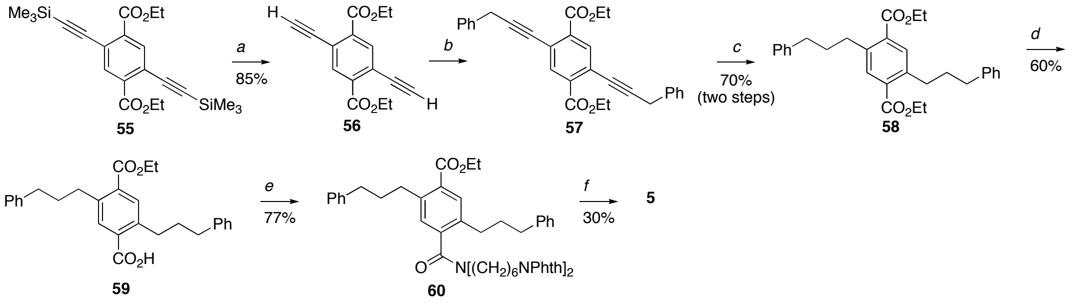

a. $AgNO_3$, Acetone:H_2O, 24h rt; *b.* $PdCl_2(CH_3CN)_2$, 2-dicyclohexylphosphino-2',4',6'-triisopropylbiphenyl, BnCl, Cs_2CO_3, THF, 65 °C, 12h; *c.* Raney Ni, H_2, MeOH, 2 h, 80% (two steps); *d.* KOH, EtOH, 80 °C, 16h; *e.* HOBt, BOP, NMM, $HN[(CH_2)_6NPhth]_2$, DMF, rt, 18h; *f.* 50% aq. NH_2OH, THF, MeOH, KCN, 80 °C, 24 h, HPLC purification using $MeCN/H_2O$/TFA.

Diethyl 2,5-diethynylterephthalate (56): To a solution of **55** [1] (0.15 g, 0.361 mmol) in acetone (10 mL) was added a solution of $AgNO_3$ (0.03 g, 0.18 mmol) in H_2O (5 mL) and the reaction mixture was stirred in dark for 24 hours at room temperature[2].The reaction mixture was poured on to a saturated brine solution (20 mL), filtered through Celite and washed with EtOAc. The layers were separated; the organic layer was dried over anhydrous $MgSO_4$ and concentrated *in vacuo*. MPLC purification of the residue using a gradient of Hex:EtOAc gave **51** as a white solid (0.08 mg, 85%).^{1}H NMR ($CDCl_3$) δ 8.14 (s, 2H), 4.40 (q, *J* = 7.2 Hz, 4H), 3.51 (s, 2H) and 1.40 (t, *J* = 7.2 Hz, 6H). ^{13}C NMR ($CDCl_3$): δ 164.73, 136.80, 135.30, 122.74, 85.12, 81.03, 62.19 and 14.37.

Diethyl 2,5-bis(3-phenylprop-1-ynyl)terephthalate (57): A disposable vial equipped with a screw cap and a stir bar was charged with $PdCl_2(CH_3CN)_2$ (2.40 mg, 0.0093 mmol), 2-dicyclohexylphosphino-2',4',6'-triisopropylbiphenyl (13.0 mg, 0.0273 mmol), and Cs_2CO_3 (0.17 g, 0.522 mmol). The vial was evacuated and back-filled with nitrogen three times. A solution of benzyl chloride (0.086 g, 0.680 mmol) and **51** (0.061 g, 0.226 mmol) in anhydrous THF (8 mL) was then added and the solution was stirred at 65 °C for 12 hours[3]. After cooling to room temperature, the reaction mixture was filtered over Celite and concentrated *in vacuo*.

The benzyl alkyne was found to be unstable during column chromatography, hence the crude reaction mixture was subjected to hydrogenation with Raney-Ni without any purification.

Diethyl 2,5-bis(3-phenylpropyl)terephthalate (**58**): A solution of crude product **57** from the above reaction in MeOH (10 mL) was added Raney Nickel (200 μL, slurry in water) and the reaction mixture was stirred at room temperature for 2 hours under a balloon of hydrogen. The reaction mixture was filtered through Celite and concentrated *in vacuo*. MPLC purification of the residue using a gradient of Hex:EtOAc gave **53** as a colorless oil (0.72 mg, 70% yield for two steps). ^{1}H NMR ($CDCl_3$) δ 7.68 (s, 2H), 7.30-7.25 (m, 4H), 7.22-7.16 (m, 6H), 4.34 (q, *J* = 7.2 Hz, 4H), 2.96 (t, *J* = 7.6 Hz, 4H), 2.69 (t, *J* = 8.0 Hz, 4H), 1.99-1.89 (m, 4H) and 1.38 (t, *J* = 6.8 Hz, 6H). ^{13}C NMR ($CDCl_3$): δ 167.62, 142.40, 141.15, 133.01, 128.67, 128.52, 125.98, 61.40, 36.09, 33.79, 33.45 and 14.53.

4-(Ethoxycarbonyl)-2,5-bis(3-phenylpropyl)benzoic acid (59): To a solution of **58** (0.05 gm, 0.109 mmol) in EtOH (10 mL) was added KOH (0.012 g, 0.218 mmol) and the reaction mixture was stirred at 80 °C for 16 hours. The reaction mixture was cooled to room temperature and the solvent was removed *in vacuo*. The residue was dissolved in water (10 mL), neutralized with 1 N HCl and extracted with EtOAc (3 x 10 mL). The combine organic extracts were dried over anhydrous $MgSO_4$, filtered and concentrated *in vacuo*. MPLC purification of the residue using a gradient of Hex:EtOAc gave **59** as a white solid (28 mg, 60%) with little impurities of the diacid. This product was carried forward to next step without any further purification.

Ethyl 4-bis(6-1(1,3-dioxoisoindolin-2-yl)hexyl)carbamoyl)-2,5-bis(3-phenylpropyl)benzoate (60): A solution of **59** (0.025 g, 0.058 mmol), **14x** (0.036 g, 0.076 mmol), BOP (0.014 mg, 0.103 mmol), HOBt (0.047 g, 0.106 mmol) and NMM (13 μL, 0.119 mmol) in anhydrous DMF (5 mL) was stirred at room temperature for 16 hours. The reaction mixture was

partitioned between water (10 mL) and CH_2Cl_2 (10 mL). The aqueous layer was extracted with CH_2Cl_2 (2 x 10 mL). The organic layers were dried over $MgSO_4$, filtered and then concentrated *in vacuo*. MPLC purification of the residue using a gradient of Hex:EtOAc gave **50** as a pale yellow oil (0.04 g, 77%).^{1}H NMR ($CDCl_3$) δ 7.84-7.81 (m, 4H), 7.71-7.69 (m, 4H), 7.26-7.22 (m, 5H), 7.17-7.13 (m, 6H), 6.99 (s, 1H), 4.32 (q, *J* = 7.2 Hz, 2H), 3.69(t, *J* = 7.2 Hz, 2H), 3.66-3.59 (m, 2H), 3.54 (t, *J* = 7.2 Hz, 2H), 3.24-3.17 (m, 2H), 2.93 (q, *J* = 7.6 Hz, 4H), 2.63 (q, *J* = 7.6 Hz, 4H), 2.60-2.53 (m, 2H), 2.02-1.91 (m, 2H), 1.91-1.85 (m, 4H), 1.73-1.69 (m, 2H), 1.64-1.54 (m, 2H), 1.54-1.47 (m, 2H) and 1.47-1.35 (m, 9H).

N^1,N^1-Bis(6-aminohexyl)-N^4-hydroxy-2,5-bis(3-phenylpropyl)terephthalamide (5): To a stirred solution of **50** (0.038 g, 0.043 mmol) in MeOH:THF (1:1, 8 mL) was added 50 % aqueous hydroxylamine (1 mL) followed by catalytic amount of KCN . The resulting mixture was stirred at 80 °C for 24 hours. After the solvent was removed *in vacuo*, the residue was subjected to HPLC purification, which gave **5.2TFA** as a colorless oil (11 mg, 30%). The semi – preparative and analytical HPLC retention time for **5•2TFA** are 15.41 (eluting time: 30 minutes) and 12.9 minutes (eluting time: 20 minutes) respectively.^{1}H NMR (CD_3OD) δ 7.81 (s, 1H), 7.28-7.23 (m, 4H), 7.20-7.12 (m, 6H), 7.05 (s, 1H), 3.67-3.62 (m, 2H), 3.39 (t, *J* = 6.8 Hz, 2H), 3.14 (t, *J* = 7.2 Hz, 2H), 3.12-2.92 (m, 4H), 2.68-2.52 (m, 6H), 2.04-1.92 (m, 2H), 1.92-1.82 (m, 2H), 1.72-1.62 (m, 4H), 1.54-1.34 (m, 8H) and 1.12-1.02 (m, 4H).

References

1. Sorensen JK, Vestergaard M, Kadziola A, Kilsa K, Nielsen MB (2006) Synthesis of Oligo(phenyleneethylene)-Tetrathiafulvalene Cruciforms for Molecular Electronics. Organic Letters 8: 1173-1176.

2. Carpita A, Mannocci L, Rossi R (2005) Silver(I)-Catalysed Protiodesilylation of 1-(Trimethylsilyl)-1-alkynes. European Journal of Organic Chemistry: 1859-1864.

3. Larsen CH, Anderson KW, Tundel RE, Buchwald SL (2006) Palladium-Catalyzed Heck Alkynylation of Benzyl Chlorides Synlett: 2941-2946.
