# Supplementary material for: Potent New Small-Molecule Inhibitor of Botulinum Neurotoxin Serotype A Endopeptidase Developed by Synthesis-Based Computer-Aided Molecular Design
Source: PLoS One. 2009 Nov 10;4(11):e7730. doi: 10.1371/journal.pone.0007730 (PMC2771286; doi:10.1371/journal.pone.0007730)
Supplement: Figure S6 — Synthesis of Analog 7. (0.07 MB PDF) [file pone.0007730.s006.pdf]

Figure S6

Synthesis of Analog 7

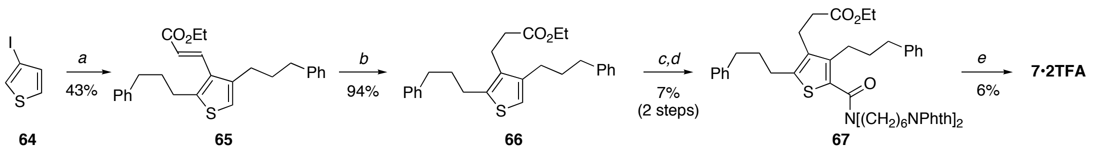

a. (3-Bromopropyl)benzene/Ethyl acrylate/$PdCl_2(PPh_3)_2$/Norbornene/Cs_2CO_3/CH_3CN, reflux, 18 h; *b.* H_2(75 psi)/Pd-C/MeOH:EtOAc (5:1),rt,14 h; *c.* $(COCl)_2$/$AlCl_3$/CH_2Cl_2,0 °C, 30min; *d.* HN[$(CH_2)_6$NPhth]$_2$/BOP/HOBt//NMM/DMF,rt,16 h; *e.* NH_2OH (aq. 50%)/ KCN/MeOH/THF, rt,16 h, HPLC purification using MeCN/H_2O/TFA.

Ethyl 3-(2,4-bis(3-phenylpropyl)thiophen-3-yl)propanoate (66). The compound **65** was prepared following the literature method [1] with a slight modification; $PdCl_2(PPh_3)_2$ was used in place of $Pd(OAc)_2$. This modification was necessary because $Pd(OAc)_2$ caused the conversion of 3-bromopropylbenzene, one of the key ingredients, to its acetate analog, resulting in not only difficulty in purification but also in lower yield. The compound **65** (124 mg, 2.96×10^{-4} mol) was dissolved in EtOAc (1 mL) and diluted with MeOH (5 mL). The resulting solution was shaken in the presence of 10% Pd-C (0.20 g) using a Parr Hydrogenation Apparatus (Moline, IL) at 75 psi for 14 hours at room temperature. The catalyst was removed by filtration through Celite, the filtrate was concentrated *in vacuo* to give **66** (118 mg, 94%). This material was used without further purification. ^{1}H NMR ($CDCl_3$) δ 7.30–7.16 (m, 10H), 6.74 (s. 1H), 4.13 (q, *J* = 7.1 Hz, 2H), 2.79–2.69 (m, 4H), 2.53 (t, *J* = 7.7 Hz, 2H), 2.37–2.33 (m, 2H), 2.00–1.92 (m, 4H), and 1.25 (t, *J* = 7.1 Hz, 3H).

Ethyl 1-(bis(6-(1,3-dioxoisoindolin-2-yl)hexyl)carbamoyl)-4-(3,5-bis(3-phenylpropyl)thiophen-3-yl)propanoate (67). A carboxylic group was introduced into the thiophene ring of the compound **65** according to a known method[2], thus to an oven dried round bottom flask was charged with anhydrous CH_2Cl_2(1 mL), followed by $AlCl_3$ (7.6 mg, 5.71×10^{-5} mol) under N_2 and then cooled to 0 °C, followed by oxalyl chloride (10 mL, 1.14×10^{-4} mol). To this was

added a solution of **66** (24 mg, 5.71×10^{-5} mol) in anhydrous CH_2Cl_2(1 mL) over 30 min. The reaction was allowed to come to room temperature, quenched with H_2O (3 mL). The layers were separated and the aqueous layer was extracted with CH_2Cl_2 (3 × 3 mL). The extracts were combined, dried over $MgSO_4$, and then filtered. To this filtrate **14x** (27 mg, 5.71×10^{-5} mol) was added, and then stirring was continued overnight. The solvent was removed *in vacuo*, the residue purified by MPLC to give **67** (4.1 mg, 7%) of the title compound. ^{1}H NMR ($CDCl_3$) δ 7.83–7.82 (m, 4H), 7.71–7.69 (m, 4H), 7.26–7.13 (m, 10H), 4.12 (q, *J* = 7.1 Hz, 2H), 3.67 (t, *J* = 7.1 Hz, 2H), 3.58 (t, *J* = 7.1 Hz, 2H), 3.38 (t, *J* = 7.5 Hz, 2H), 3.16 (t, *J* = 7.7 Hz, 2H), 2.92 (m, 2H), 2.81–2.67 (m, 6H), 2.28 (t, *J* = 8.1 Hz, 2H), 1.98 (t, *J* = 7.5 Hz, 2H), 1.82 (m, 2H), 1.69–1.57 (m, 8H), 1.40–1.34 (m, 6H), and 1.27–1.23 (m, 11H).

N,N-Bis(6-aminohexyl)-4-(3-(hydroxyamino)-3-oxopropyl)-3,5-bis(3-phenylpropyl)thiophene-2-carboxamide (7). The compound obtained above (11.7 mg, 1.27×10^{-5} mol) was dissolved in MeOH :THF (0.5 mL each), followed by 50% aqueous NH_2OH (0.5 mL) and one crystal of KCN, and then at room temperature for 16 hours. The reaction was monitored by analytical HPLC (Phenomenex Gemini 4.6 × 250 mm, 5 m, C18, linear gradient of 20% B to 100% B over 20 min, solvent A = 1000 mL H_2O and 1 mL TFA; solvent B = 900 mL H_2O, 100 mL MeCN, and 1 mL TFA), to give **7.2TFA** (0.74 mg, 6%). ^{1}H NMR (400 MHz, CD_3OD) δ 7.28–7.16 (m, 10H), 3.44 (t, *J* = 7.3 Hz, 2H), 3.25 (t, *J* = 7.6 Hz, 2H), 3.13–2.98 (m, 2H), 2.96–2.90 (m, 4H), 2.79–2.69 (m, 8H), 2.16 (m, 2H), 1.98 (t, *J* = 7.1 Hz, 2H), 1.82 (m, 2H), and 1.69–1.20 (m, 16H).

References

1. Mitsudo K, Thansandote P, Wilhelm T, Mariampillai B, Lautens M (2006) Selectively Substituted Thiophenes and Indoles by a Tandem Palladium-Catalyzed Multicomponent Reaction. Organic Letters 8: 3939-3942.

2. Neubert ME, Fishel DE (1990) Preparation of 4-Alkyl-and 4-Halobenzoyl Chlorides: 4-Pentylbenzoyl Chloride. Organic Syntheses Collective Volume 7: 420.
