# Supplementary figures and images for: Potent New Small-Molecule Inhibitor of Botulinum Neurotoxin Serotype A Endopeptidase Developed by Synthesis-Based Computer-Aided Molecular Design
Source: PLoS One. 2009 Nov 10;4(11):e7730. doi: 10.1371/journal.pone.0007730 (PMC2771286; doi:10.1371/journal.pone.0007730)

# 8 before HPLC

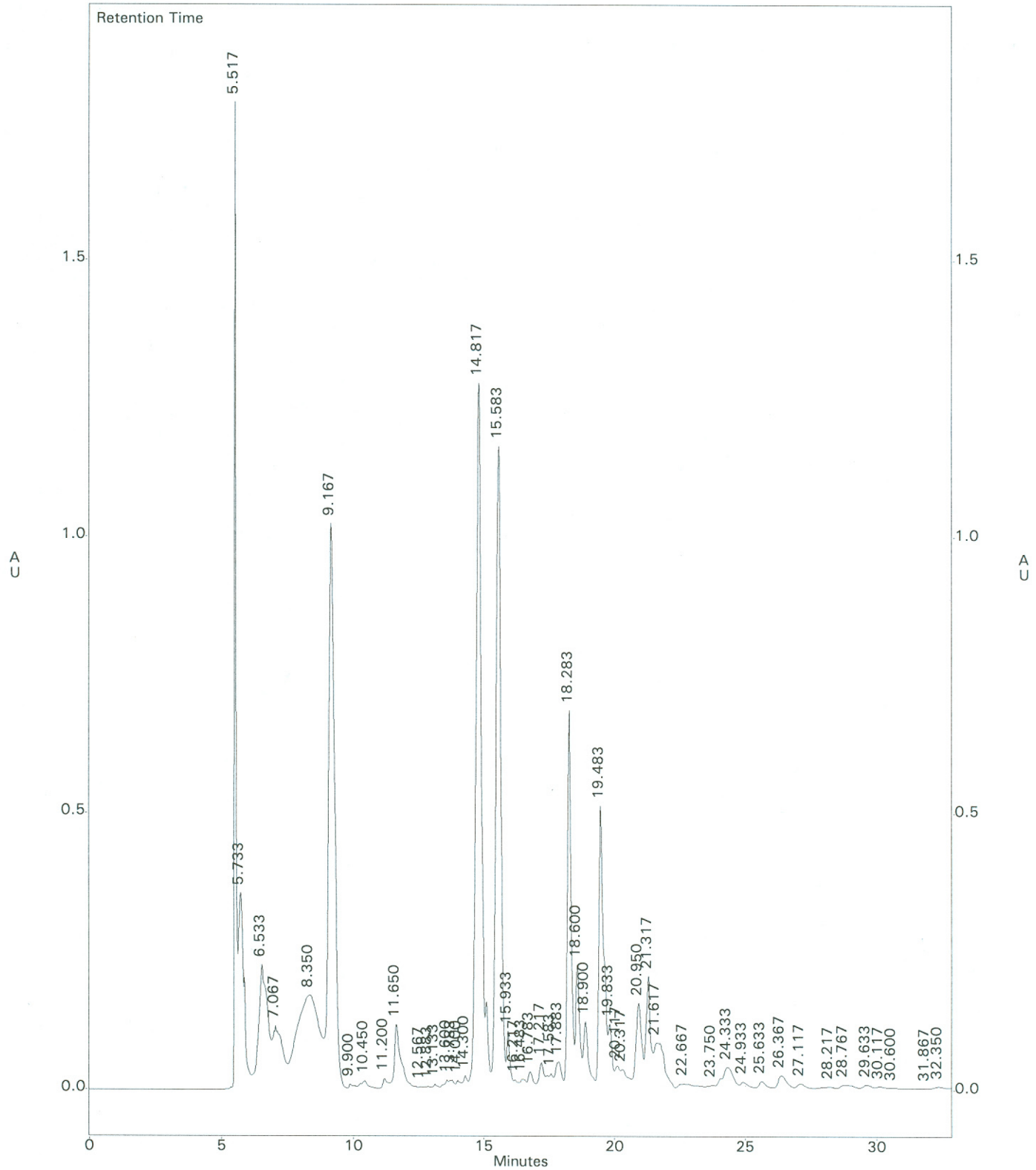

# 8 after HPLC

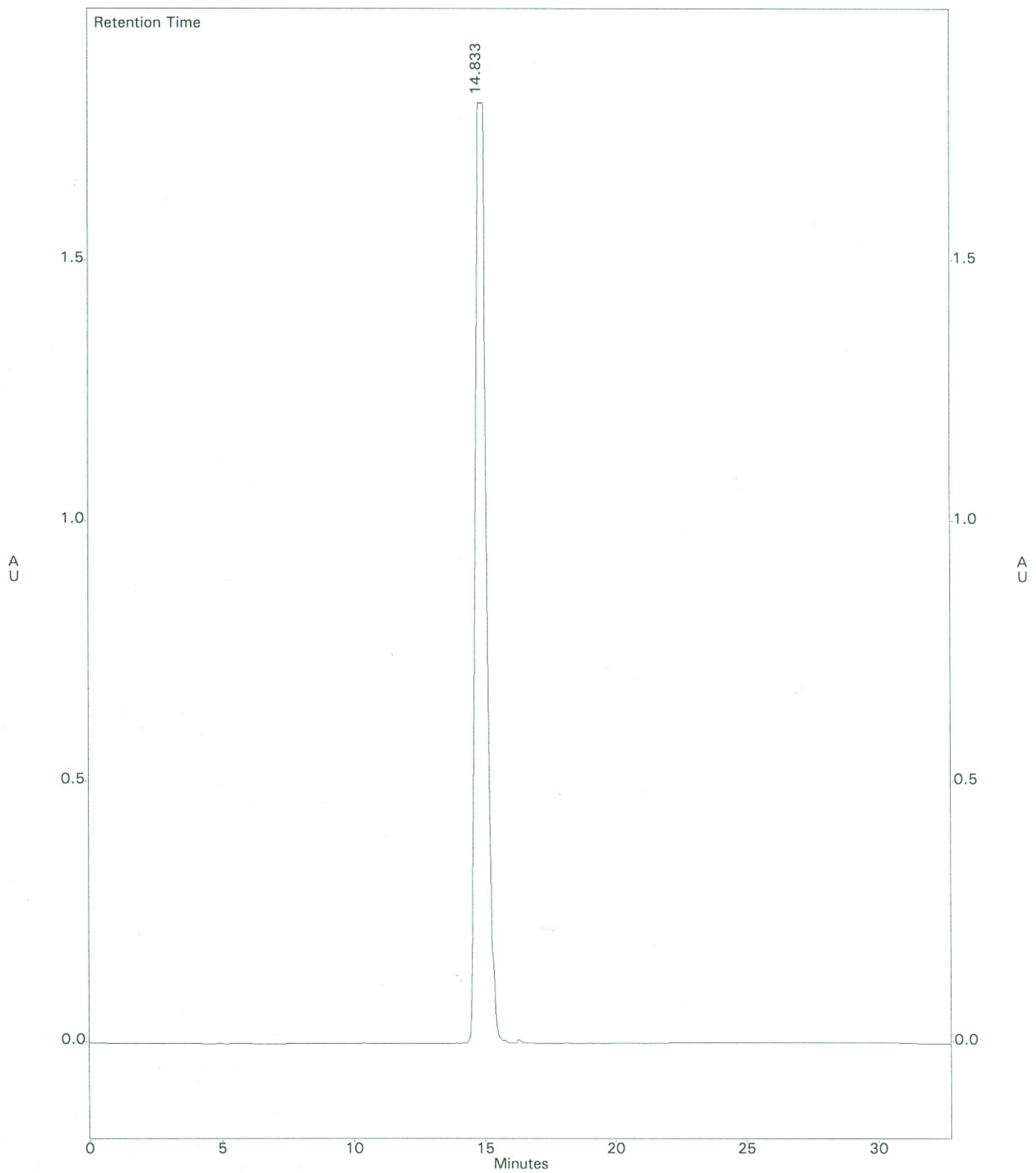

Supplement: Figure S7 — Chromatograms of 8•2TFA before and after the HPLC purification. (6.12 MB PDF) [file pone.0007730.s007.pdf]

# 9 before HPLC

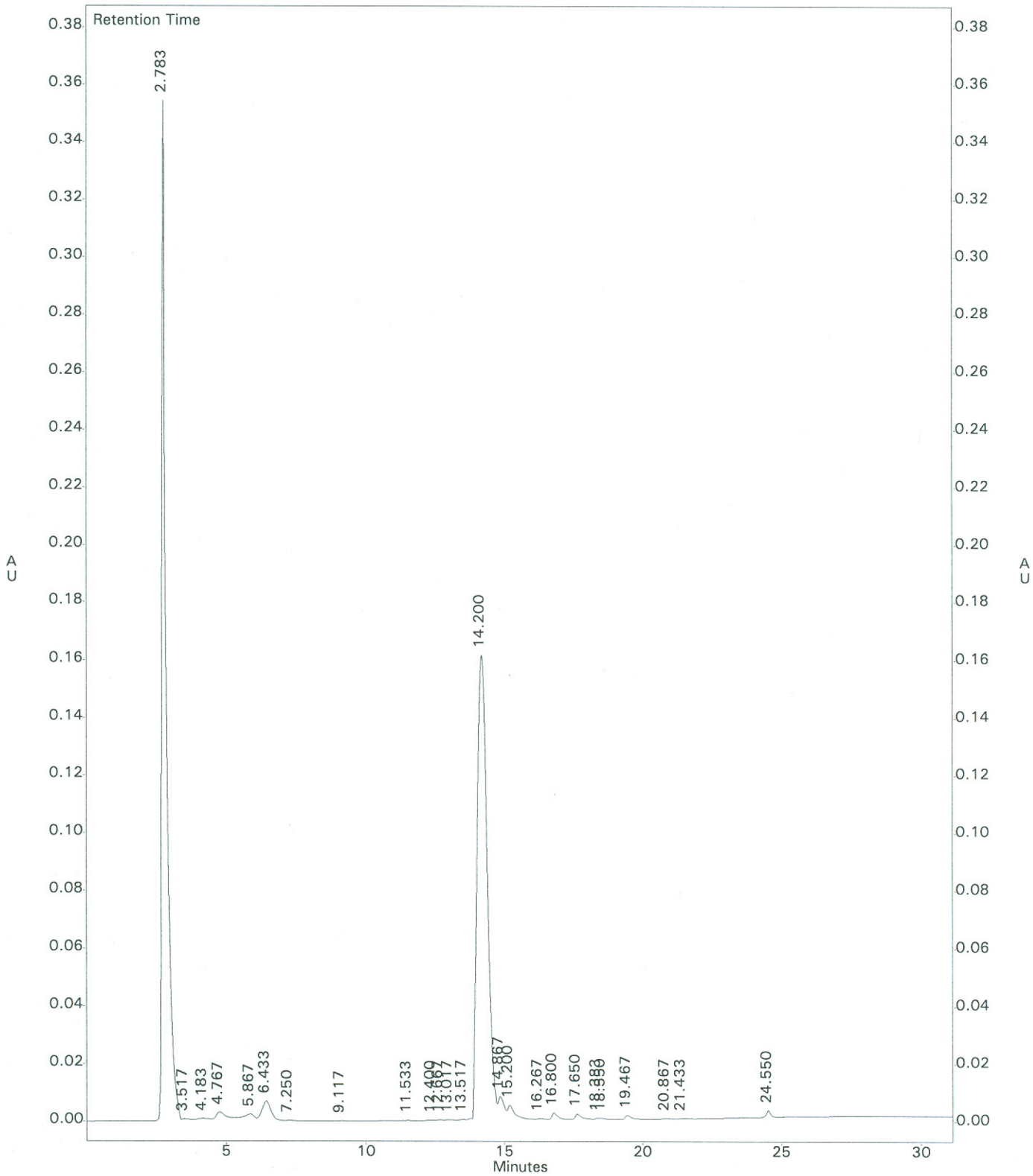

# 9 after HPLC

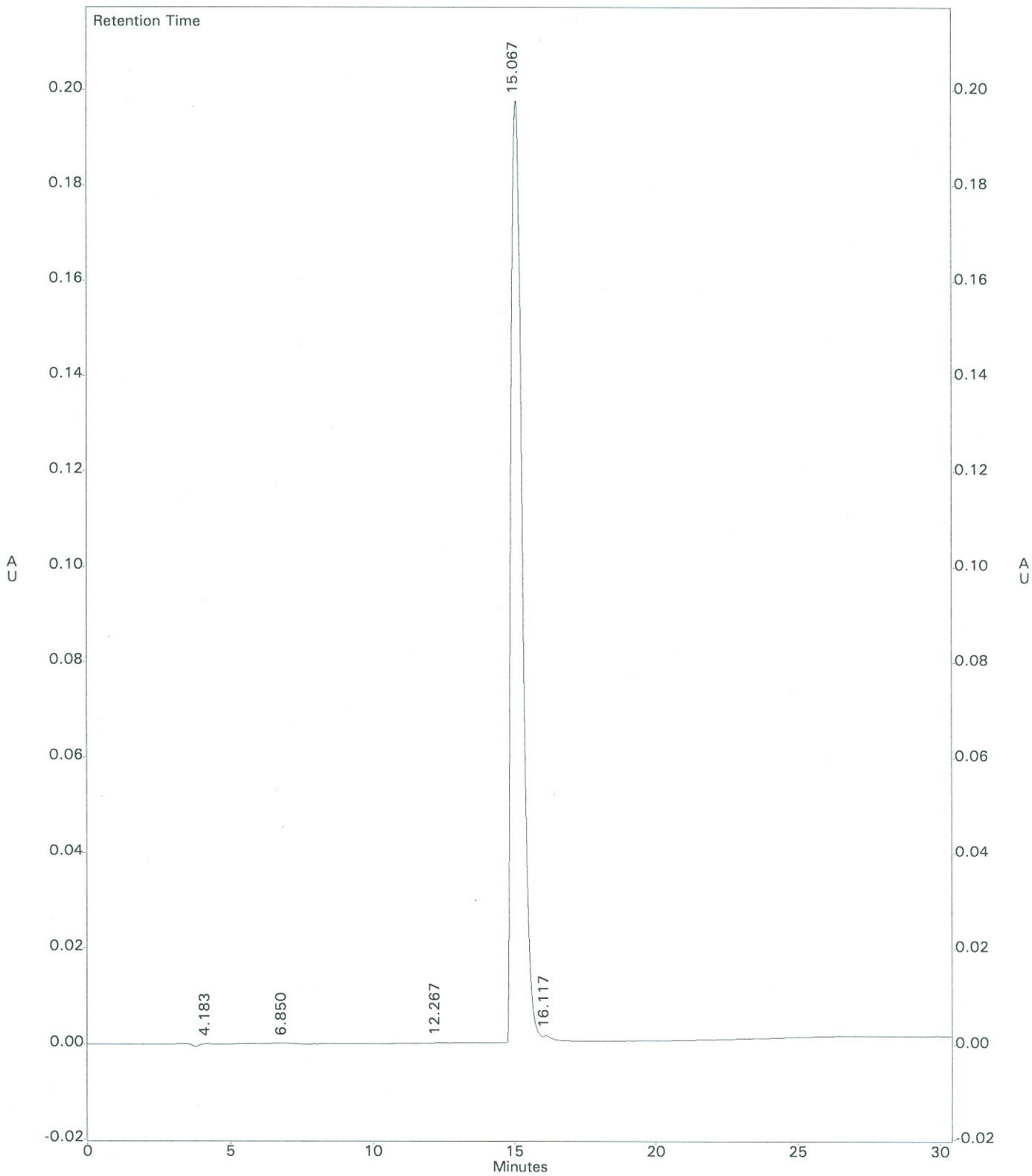

Supplement: Figure S8 — Chromatograms of 9•2TFA before and after the HPLC purification. (6.17 MB PDF) [file pone.0007730.s008.pdf]

# 10b before HPLC

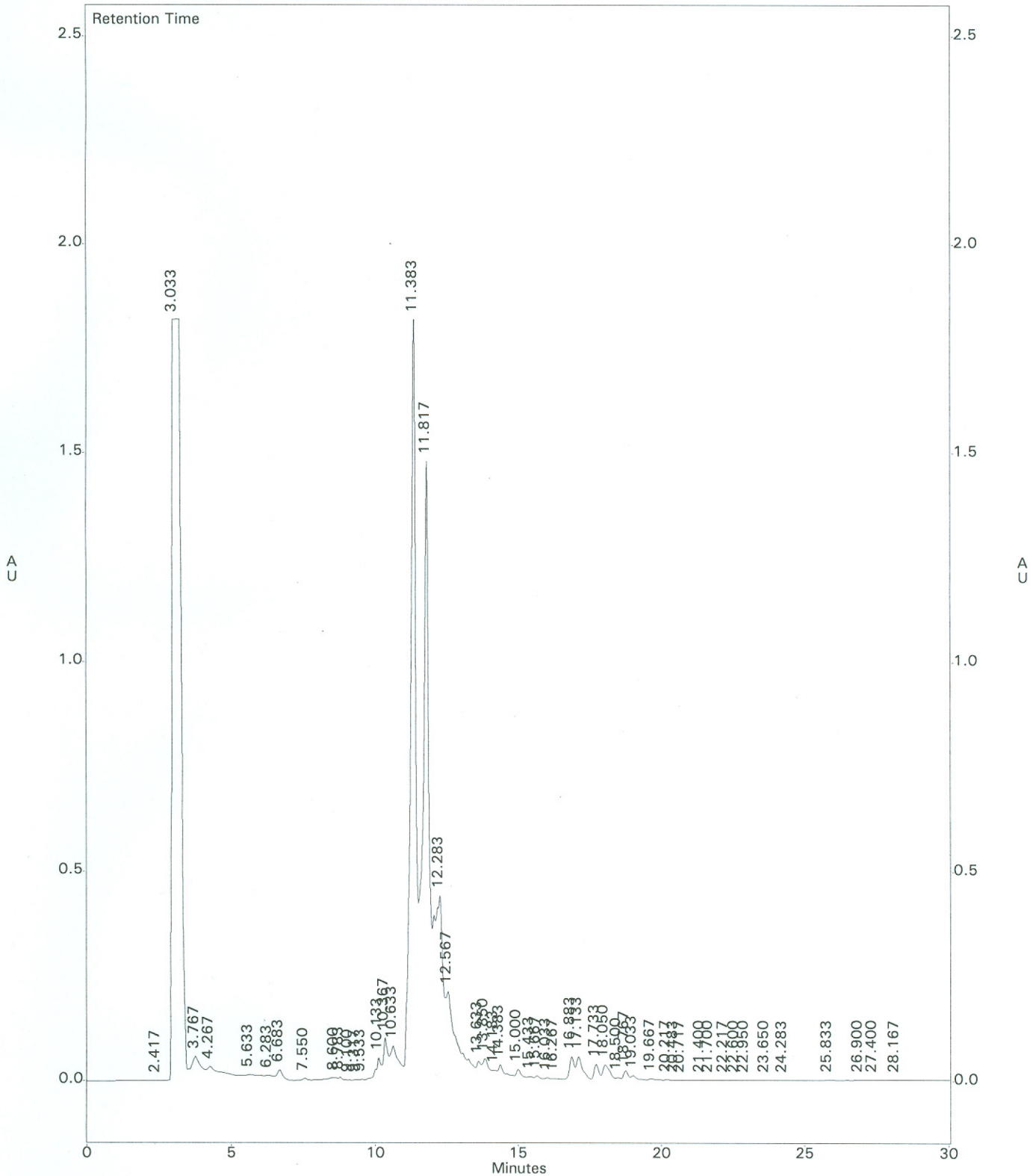

# 10b after HPLC

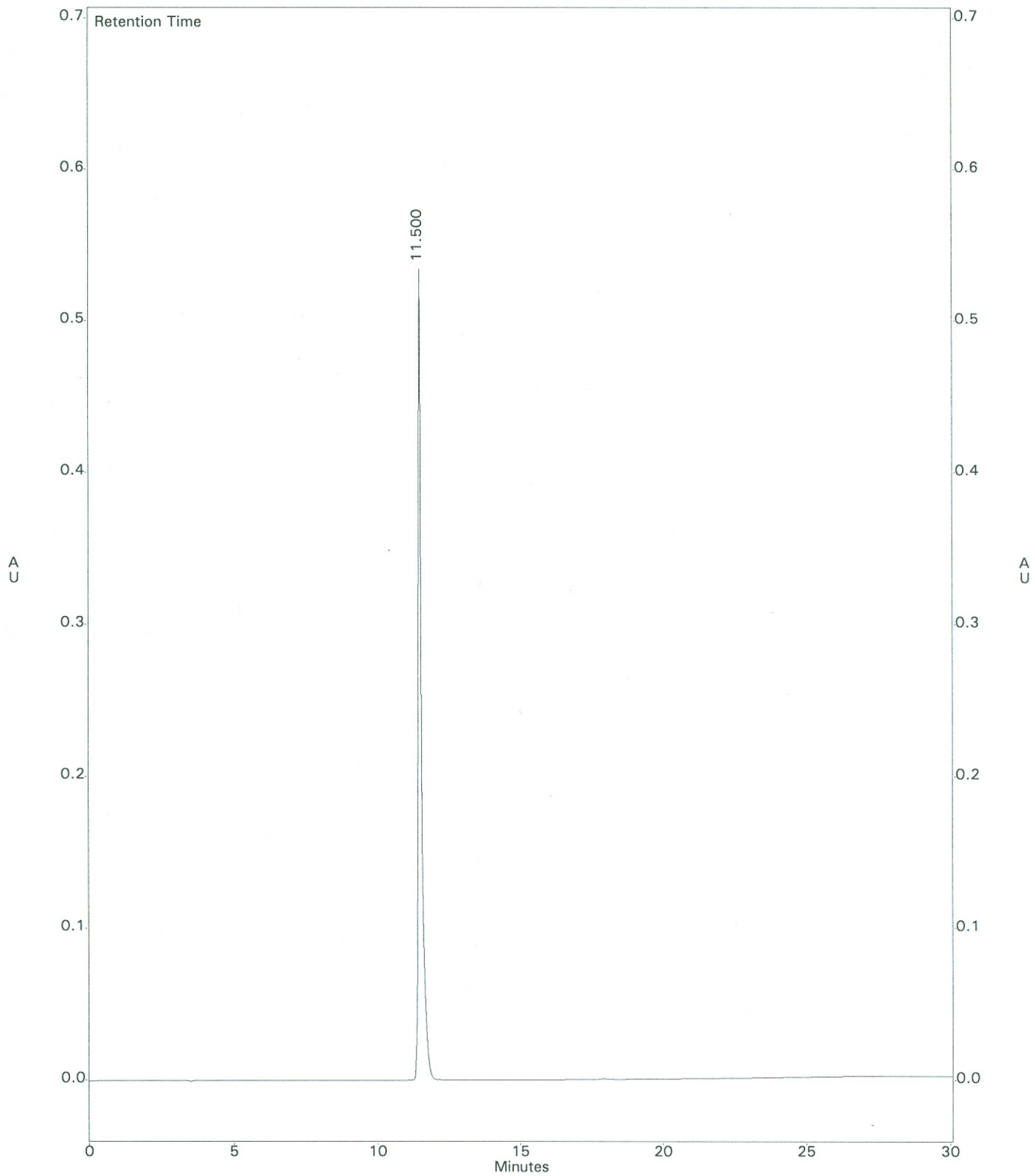

Supplement: Figure S9 — Chromatograms of 10b•2TFA before and after the HPLC purification. (6.11 MB PDF) [file pone.0007730.s009.pdf]

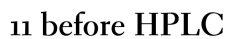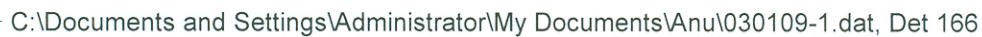

# 11 after HPLC

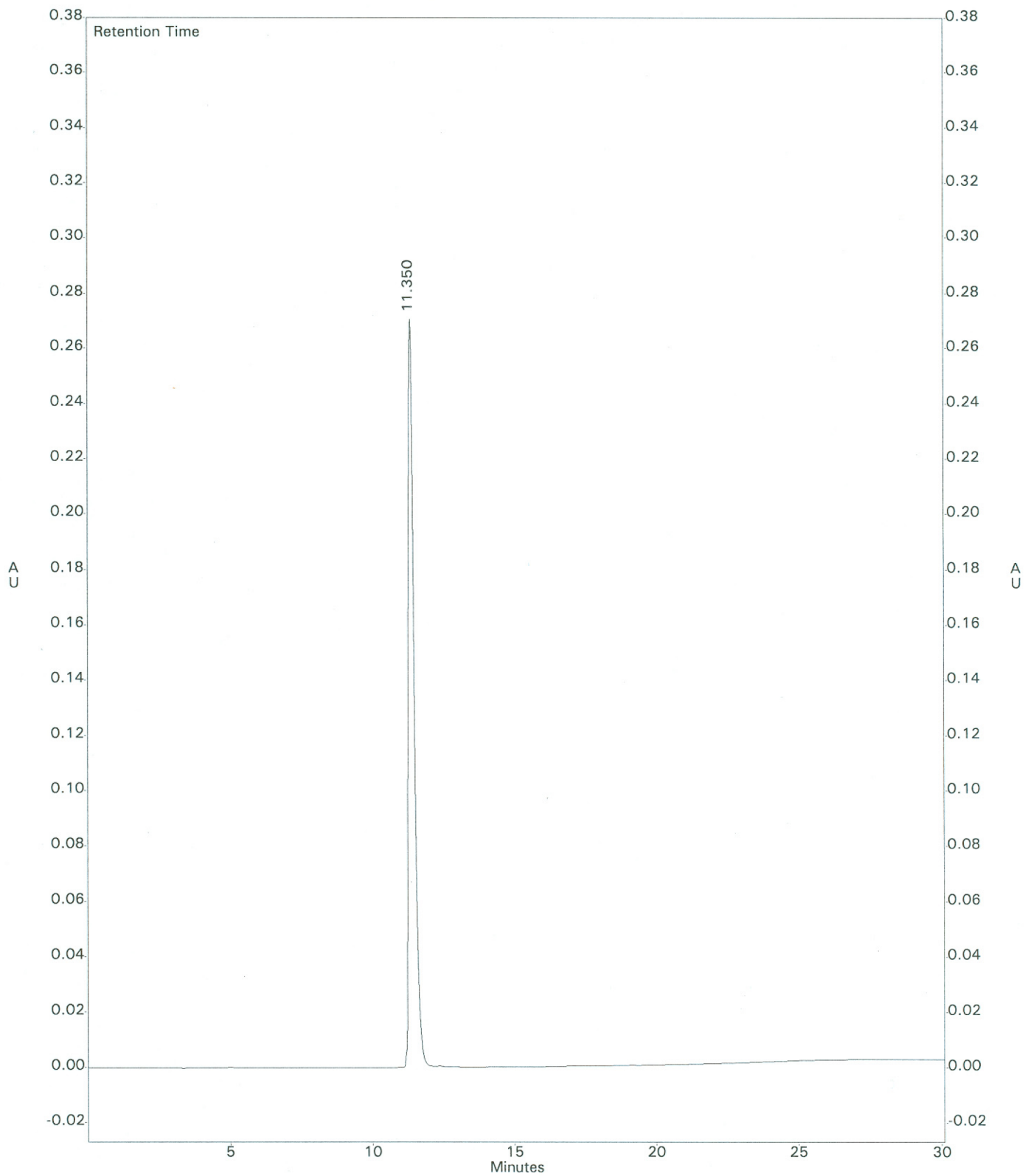

Supplement: Figure S10 — Chromatograms of 11•2TFA before and after the HPLC purification. (6.45 MB PDF) [file pone.0007730.s010.pdf]

# AHP before HPLC

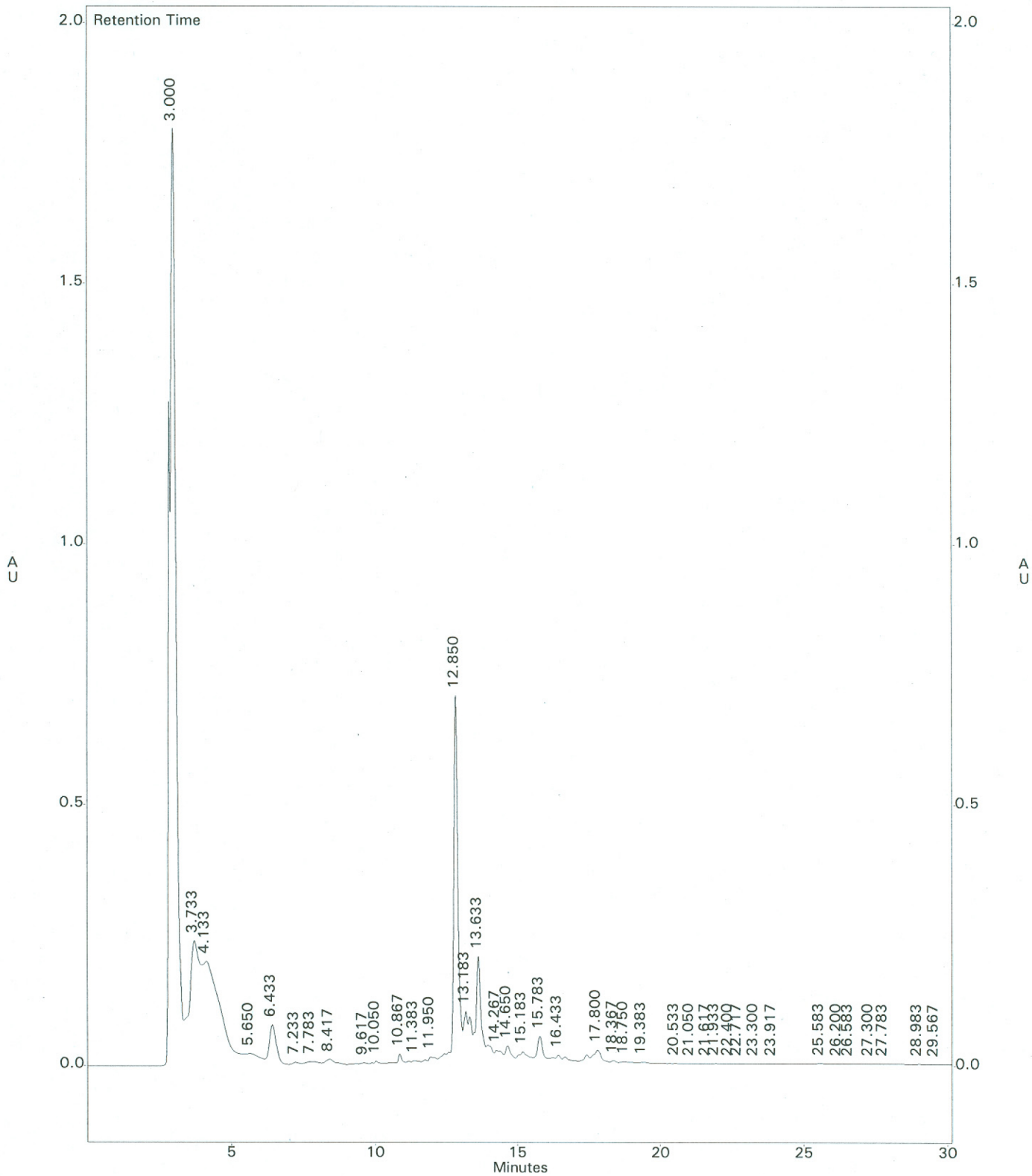

# AHP after HPLC

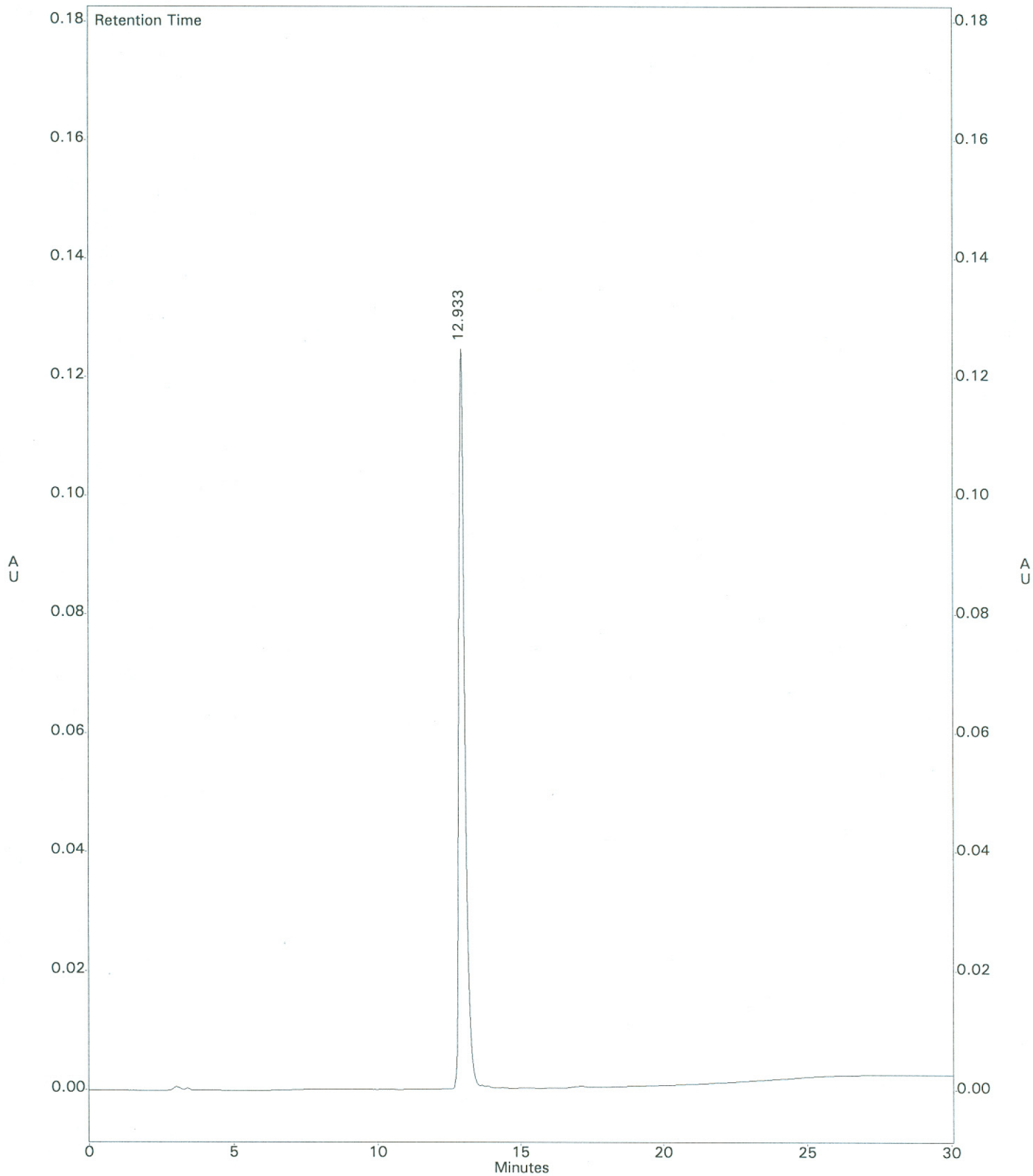

Supplement: Figure S11 — Chromatograms of AHP•2TFA before and after the HPLC purification. (6.03 MB PDF) [file pone.0007730.s011.pdf]
